# Trichome density and herbivore behaviour on tomato is influenced by herbivory, plant age, and leaf surface

**DOI:** 10.1093/aobpla/plaf057

**Published:** 2025-10-07

**Authors:** Sunil Aryal, Manish Gautam, Justin George, Gadi V P Reddy, Rupesh Kariyat

**Affiliations:** Department of Entomology and Plant Pathology, University of Arkansas, Fayetteville, AR, United States; Nepal Agricultural Research Council, Singhadurbar Plaza, Kathmandu, Nepal; Department of Entomology and Plant Pathology, University of Arkansas, Fayetteville, AR, United States; USDA-ARS, Southern Insect Management Research Unit, Stoneville, MS 38776, United States; USDA-ARS, Southern Insect Management Research Unit, Stoneville, MS 38776, United States; Department of Entomology and Plant Pathology, University of Arkansas, Fayetteville, AR, United States

**Keywords:** Type VI trichome, non-glandular trichome, *Spodoptera exigua*, adaxial surface, abaxial surface, feeding initiation

## Abstract

Leaf trichomes in plants act as the first line of physical defence against herbivory, in addition to many other reported functions. Although trichomes have been found to vary intraspecifically and can be induced by herbivory, their interactive effects under additional factors, such as plant age and abaxial vs. adaxial leaf surfaces, are less understood. Here, using five common tomato varieties, we explored the effects of these factors and their interactions on trichome type and density. We quantified the densities of Type VI glandular trichomes, non-glandular trichomes, and total trichomes on abaxial and adaxial leaf surfaces, and total leaf trichomes with and without herbivory by *Spodoptera exigua* at both vegetative and reproductive stages. Further, we also tested whether the time taken to initiate feeding by *S. exigua* larvae could be influenced by the number of trichomes on the adaxial and abaxial surfaces. The results showed that there is significant variation in trichome density among varieties and leaf surfaces. Also, there were differences in herbivory-induced trichome production, with variable responses across varieties and growth stages. Bioassay results showed that insects took longer to initiate feeding on the abaxial leaf surface than on the adaxial surface, potentially due to the higher density of non-glandular trichomes on the abaxial side. Collectively, we report that the regulation and development of trichomes on the leaf surface of tomatoes is governed by multiple factors, with potential consequences for herbivore feeding, suggesting how physical defences play a significant role in insect–plant interactions.

## Teaser

Leaf trichomes are commonly considered as the first line of plant defense against insect herbivory. In this study, using 5 tomato varieties, we show that in addition to varietal differences, leaf side also influences trichome density. And, larvae of Spodoptera exigua took significantly longer to feed on the leaf side that had higher trichome density, demonstrating the overall effects of tomato trichomes against herbivory.

## Introduction

Plant defence mechanisms against biotic and abiotic stressors consist of various morphological, biochemical, and molecular processes (War et al. 2012, Mitchell et al. 2016). Foliar pubescence, or leaf trichomes, is one of the morphological characteristics that have multiple functions in plants (Karabourniotis et al. 2020). Leaf trichomes have evolved in plants as the first line of physical defence against myriad of stressors, including herbivory (Tian et al. 2012). Trichomes not only act as physical barriers restricting the herbivores’ movement (Voigt and Gorb 2010) but also cause adverse effects on insect feeding, growth, and development (Eigenbrode et al. 1996, Kaur and Kariyat 2023). Owing to their inducible nature and response against insect herbivores, trichomes have been extensively studied in several agricultural crops, including economically important crops like tomato (*Solanum lycopersicum*). In *Solanum* spp., trichomes have been characterized and classified into seven distinct types: Types I, IV, VI, and VII as glandular and Types II, III, and V as non-glandular trichomes (NGTs) (Wilkens et al. 1996, Simmons and Gurr 2005). Recently, additional trichome types have been identified, and 9 types of trichomes have been described based on a study of 10 tomato varieties (Kaur et al. 2023 ). Due to their differences in shapes, sizes and functions, different types of trichomes can confer varying degrees of defences against herbivores in crops.

Recent advances in the study of trichomes have presented evidence to show that both glandular trichome and non-glandular trichome have a critical role in driving the host–herbivore interactions. Glandular trichomes aid via chemical defences (Wilkens et al. 1996 , Tian et al. 2012), whereas NGTs act as physical barriers against herbivores (Gairola et al. 2009 , Khederi et al. 2014 ). For example, Neiva et al. (2013) showed a negative relationship between glandular trichome and resistance to whiteflies in tomatoes. Similarly, the survival of neonate beet armyworm (*Spodoptera exigua*), a major herbivore of solanaceous crops, was also negatively affected by glandular trichomes of tomatoes (Eigenbrode and Trumble 1993). In another study, NGTs in soybean (*Glycine max*) have been found to delay feeding initiation in fall armyworm (*Spodoptera frugiperda*) and soybean looper, *Chrysodeixis includens* (Ayala et al. 2024) showing both pre- and post-ingestive effects. NGTs that are hooked can even arrest the movement of the caterpillars by piercing their cuticle and causing loss of blood, eventually leading to their death (Chowdhury et al. 2025). However, each trichome type can vary distinctly in their number per unit leaf area (trichome density) in plants; in tomatoes, trichomes have been found to vary in their densities across both adaxial and abaxial surfaces of the leaves (Oney and Bingham 2014, Paudel et al. 2019). Collectively, most studies show that dense trichomes provide defence against multiple herbivores in wild and cultivated solanaceous species (Nord et al. 2020, Gyawali et al. 2021, Zeist et al. 2021).

Such defences, however, may vary according to the types of trichomes present on adaxial and abaxial surfaces of the leaves. For example, a higher density of NGTs on the abaxial side has been found to impede larvae from commencing feeding on the leaves (Kariyat et al. 2018, Watts and Kariyat 2021a). Effects of trichomes on herbivores can be dependent on trichome type. For example, Kwon et al. (2006) found that higher densities of long trichomes reduced *S. exigua* infestation in potato (*Solanum tubersosum*), whereas higher densities of short trichomes were linked to higher infestation levels. Trichome production can change in response to herbivory, with effects sometimes transcending generations (Agrawal 2001, Hagenbucher et al. 2013, Kaur and Kariyat 2020). However, such effects may vary across abaxial and adaxial sides. For instance, Oney and Bingham (2014) showed that trichome density on the adaxial surface was significantly increased under natural herbivory in tomato plants. Clearly, the density of trichomes can significantly determine the degree of effectiveness posed by each trichome type towards host resistance/tolerance against the herbivores. However, these are also dependent on the varieties as well as their phenological stages.

Inherently, different varieties can possess several variations of trichomes in terms of their types and density and may confer varying degrees of resistance to insect herbivores. Tomato varieties with denser trichomes (glandular and NG) have been reported to have enhanced resistance against several herbivores (Maluf et al. 2007, de Souza Marinke et al. 2022). Even among the varieties, trichomes can differ in their densities depending on the leaves’ ontogeny and phenological stages. It has been shown that new apical leaves have denser trichomes than expanded leaves in the vegetative stage, but they were almost similar at the reproductive stage in six tomato varieties (Mymko and Avila-Sakar 2019). Similarly, Gairola et al. (2009) found that young tomato leaves were densely covered with trichomes, which decreased progressively as the leaves matured. But a contrasting observation in *Turnera velutina* indicates that trichomes increased in density with the ontogeny of the plants (Ochoa-López et al. 2015). A recent study in soybean showed that trichome density was lower in the reproductive stage compared with the vegetative stage (Gandham et al. 2024). These findings suggest the highly dynamic nature of trichomes that can be influenced by various factors such as genotypes, phenological stages, leaf surfaces, and herbivory.

However, in most studies, these factors have been examined either individually or in limited combination with others. Specifically, the effect of the phenological stage, abaxial vs. adaxial leaf surfaces, variety, and herbivory damage as major driving factors of trichome production in tomatoes and their interactive effects are less understood. Therefore, we used five tomato varieties commonly grown in the United States to comprehensively examine the impact of phenological stage (vegetative and reproductive), leaf surfaces (abaxial and adaxial), and their interactions on glandular trichome and NGT during the presence and absence of herbivory by beet armyworm, *S. exigua* (Hübner). We expected that the glandular and NGT density would differ according to the phenological stages and herbivory treatment since both trichome types require differential investment of resources according to the stages and the conditions they are in. We also hypothesized that varieties will significantly differ in terms of the trichome density for each type. Furthermore, we also evaluated the time taken to initiate feeding by *S. exigua* larvae, a common herbivore of tomato, to understand how trichomes impede larval feeding of tomato leaves (Kariyat et al. 2017a). And we hypothesized that *S. exigua* larvae would be significantly delayed by the presence of denser trichomes. By including all these important factors (isolated and interactive effects) affecting trichomes, we present a holistic approach to study trichomes in tomato crops, demonstrating the importance of including trichomes as a trait of interest in building resilient crops.

## Materials and methods

### Insect and plant

*Spodoptera exigua* eggs were purchased from Frontier Agricultural Science, Newark, Delaware. They were hatched and reared on a prepared diet as per the supplier protocol (Frontier Agricultural Science, Newark, Delaware) in the laboratory with 25°C ± 1°C and 65% ± 10% RH at the University of Arkansas until being used for this experiment. Adults were reared in the laboratory on an artificial diet, and the egg-laying surface was provided in a container, as described by Lalitha and Ballal (2015). Seeds of five tomato varieties (Table 1) were purchased from Johnny's Selected Seeds (https://www.johnnyseeds.com). Varieties were selected based on their genetic and phenotypic trait diversity, such as vigour, leaf type, fruit type, and seed type. These varieties are commonly grown commercial cultivars and are disease resistant. Plants were grown in deep square plastic pots (14 cm deep) filled with soil (Pro-Mix LP 15, Premier Tech) with a regular supply of irrigation and fertilizer. The greenhouse average minimum and maximum temperature (°C), minimum and maximum relative humidity (%) and light radiation (µmol m⁻^2^ s⁻^1^) maintained during the study period were min 19.94 ± 0.12°C, max 26.2 ± 0.32°C, min 28.45 ± 2.10%, max 52.33 ± 2.04% and 162.49 ± 17.13 (µmol) m⁻^2^ s⁻^1^, respectively.

**Table 1.** Characteristics of tomato varieties used in the study.

Variety	Fruit type	Seed type	#Days to maturity	Leaf type	Plant type
Amish Paste Organic	Heirloom plum	OP	85	Narrow	Indeterminate
Big Beef F1	Beefsteak	Hybrid	70	Narrow	Indeterminate
Celebrity Plus F1	Slicing tomato	Hybrid	78	Normal	Determinate
Nepal Organic	Heirloom	OP	78	Narrow	Indeterminate
Supersweet 100 F1	Cherry	Hybrid	60–70	Normal	Indeterminate

Sources: https://njaes.rutgers.edu/tomato-varieties/, https://tomatogrowers.com/pages/tomatoes, www.johnnyseeds.com/vegetables/tomatoes/. OP represents open-pollinated varieties. Indeterminate plant types grow continuously and keep producing flowers and fruits while determinate varieties stop growing at a certain height and produce fruits for a short period. Number of days to maturity is the duration required for the varieties from planting a seed or transplanting to the harvest stage.

### Herbivory and trichome study

Constitutive and induced leaf trichomes were estimated for five varieties. Herbivory was imposed by releasing a single fourth instar *S. exigua* in an organza bag and allowing to feed on a single compound leaf for 72 h at vegetative and reproductive stages of tomato plants. During the vegetative stage, all the varieties were at 3 weeks of age (five to six leaf stages). In the reproductive stage, due to variation in maturity time among varieties, herbivory was administered at 8 weeks (8–10 leaf stage) for Big Beef H1 and Supersweet 100, and at 10 weeks (10–12 leaf stages) for Amish Paste Organic, Celebrity Plus F1 and Nepal Organic. In both stages, *S.* e*xigua* were allowed to feed on the third fully expanded leaf from the top of the plant.

Trichome estimation was performed on three apical leaflets from newly emerged topmost compound leaves (Chen et al. 2018, Mymko and Avila-Sakar 2019). We randomly selected six plants during the vegetative stage (three leaflets per plant; *n* = 18 leaflet) and five during the reproductive stage (three leaflets per plant; *n* = 15 leaflet) including both herbivore-induced and control plants. The topmost newly opened compound leaves were excised from all varieties and transported to the laboratory for trichome estimation. Trichome counts were completed within a span of 2–3 days on both the adaxial and abaxial surfaces for all varieties and treatments. The petioles of the leaves were submerged in water to maintain their turgor. Leaf discs were obtained from the collected leaflets using a 0.5-in. hole puncher and observed under 10× compound microscopy (Carl Zeiss Axiolab RE, Germany) with a field of view area of 0.86 mm^2^ (Balakrishnan et al. 2024, Gautam and Kariyat 2025a ). The number of trichomes was counted within the field of view to obtain the trichome density per 0.86 mm^2^ area for further analysis and data representation. The collected leaf samples were similar in dimension and potential differences in leaf expansion were minimized by using leaves of same age and position in the plant. To exclude the main vein from our observations, leaf discs were punched from the central region of each leaflet, between the main vein and the leaf edge (Leite et al. 2001). Trichome estimation was conducted for both adaxial and abaxial leaf surfaces.

To estimate the effects of herbivory on trichome induction, trichomes were estimated 2 weeks after the herbivory treatment (Boughton et al. 2005). The constitutive and induced trichomes were counted and categorized as Type VI trichomes, NGTs, and other glandular trichomes. Additionally, Type VII (glandular) trichomes, which were low in number, were also included in the total trichome count for analysis. Trichomes were observed for clear differentiation between the different types by using a desktop scanning electron microscope (DSEM; SNE-4500 Plus Tabletop Nanoimages LL, Pleasanton, CA, USA). A leaf disc was obtained by using a hole puncher and was mounted on the aluminium stubs with carbon tape and inserted in the SEM for scanning and imaging. No chemical treatment and sputter coating was needed for the leaf disc (Watts and Kariyat 2021).

### Estimation of feeding initiation

To assess the effect of leaf trichomes on feeding initiation by *S. exigua*, first instar larvae (*n* = 20 per side, per variety) were released on the abaxial or adaxial surface of leaves obtained from all five varieties at vegetative and reproductive stages. Each larva was observed under a handheld digital microscope (TAKMYL, Amazon, China) attached to a PC (Ayala et al. 2024), and the time to initiate feeding was recorded for up to 5 min where the larva failed to initiate feeding within 5 min were discarded. A stopwatch was used to record the time in seconds between the larva's placement and commencement of feeding. The larvae were starved for 2 h before placing them onto leaf surfaces. Feeding by the larvae was confirmed by observing feeding punctures on the leaf. To standardize the observation, a single observer recorded the time (seconds) for the caterpillars to take the first bite of the leaf.

### Statistical analysis

This study included four main factors: herbivory treatment (control and herbivory), varieties (five cultivated tomato varieties), leaf surface (adaxial and abaxial surfaces), and phenological stages (vegetative and reproductive). The trichome density was estimated for Type VI glandular trichomes, and NGTs for each treatment. For obtaining the total leaf trichome density, all the types of trichomes were added from both adaxial and abaxial surface. The effects of all four factors (independently and under interactions with each other) were analysed using generalized linear model (GLM) with poison distribution initially but replaced by negative binomial regression model since Poisson distribution exhibited a high overdispersion ratio. Additionally, GLM with a log link function and a gamma distribution was used to analyse the time (seconds) to initiate feeding data to model the relationship between the tomato varieties, leaf surface (adaxial and abaxial), and plant phenological stages (vegetative and reproductive). GLM with log link function uses the formula as:


$$\log\;(\mu )=\beta_{0}+\beta_{1}x_{1}+\beta_{2}x_{2}+\cdots +\beta_{k}x_{k}$$

where *μ* represents the expected value of the response variable; *β*_0_ is the intercept coefficient; *β*_1_, *β*_2_, …, *β_k_* are the regression coefficients associated with each predictor.

The independent effects of herbivory treatment were analysed by pooling the data across all other treatments, and the same was followed to test the isolated effects of variety, phenological stages, and leaf surface. The means were compared using Tukey’s *post hoc* test at 0.05% alpha level. The analysis was performed using R studio (R Core Team 2023) with *glm.nb* function, and visualizations (box plot) were created with ggplot2 (version 3.5.1).

### Ethics approval

The species used in this experiment are not endangered or protected.

### Consent for publication

All authors consent for publication.

## Results

### Scanning electron micrographs of trichomes on the leaf surface

**Figure 1.**
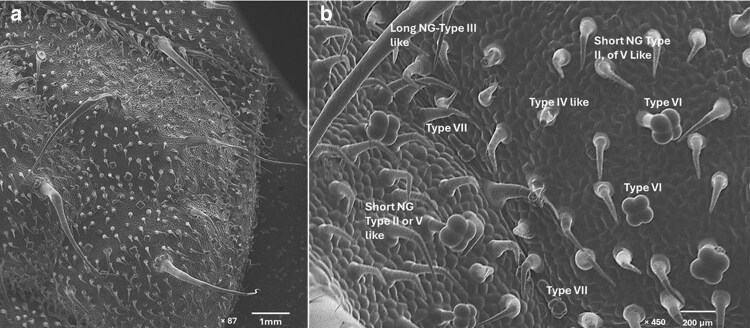
Observation of different trichome types on a newly emerged adaxial leaf surface of tomato. a) General composition of adaxial leaf surface showing different types of trichomes (×87 magnification) under SEM. b) Different types of glandular (Types IV-like, VI, and VII) and NGTs (Types II, III, and V-like) (×450 magnification) under SEM. Images were taken from leaves of Super Sweet 100 variety by using a DSEM (SNE-4500 Plus Tabletop Nanoimages LL, Pleasanton, CA, USA).

The adaxial surface of the tomato leaf under observation consisted of short NG-Type II-like, long NG-Type III-like, Type IV, Type V-like, Type VI, Type VII, and Type VIII trichomes (Fig. 1a and b). Type VI glandular trichomes are short (∼0.1 mm) with four glandular cells at the top (Glas et al. 2012, Bergau et al. 2015) . Likewise, another glandular trichome (Type VII) are very short ∼0.05 mm having four to eight cells (Glas et al. 2012). Among the NG types, Type V lacks glandular cells (Glas et al. 2012), Type III trichomes are thin, and long measuring 0.4–1.0 mm in length with a unicellular flat base (Channarayappa et al. 1992), whereas Type II trichomes are shorter (0.2–1.0 mm) with a globular multicellular base (Channarayappa et al. 1992, Glas et al. 2012).

### Trichome densities

#### Type VI glandular trichomes

**Figure 2.**
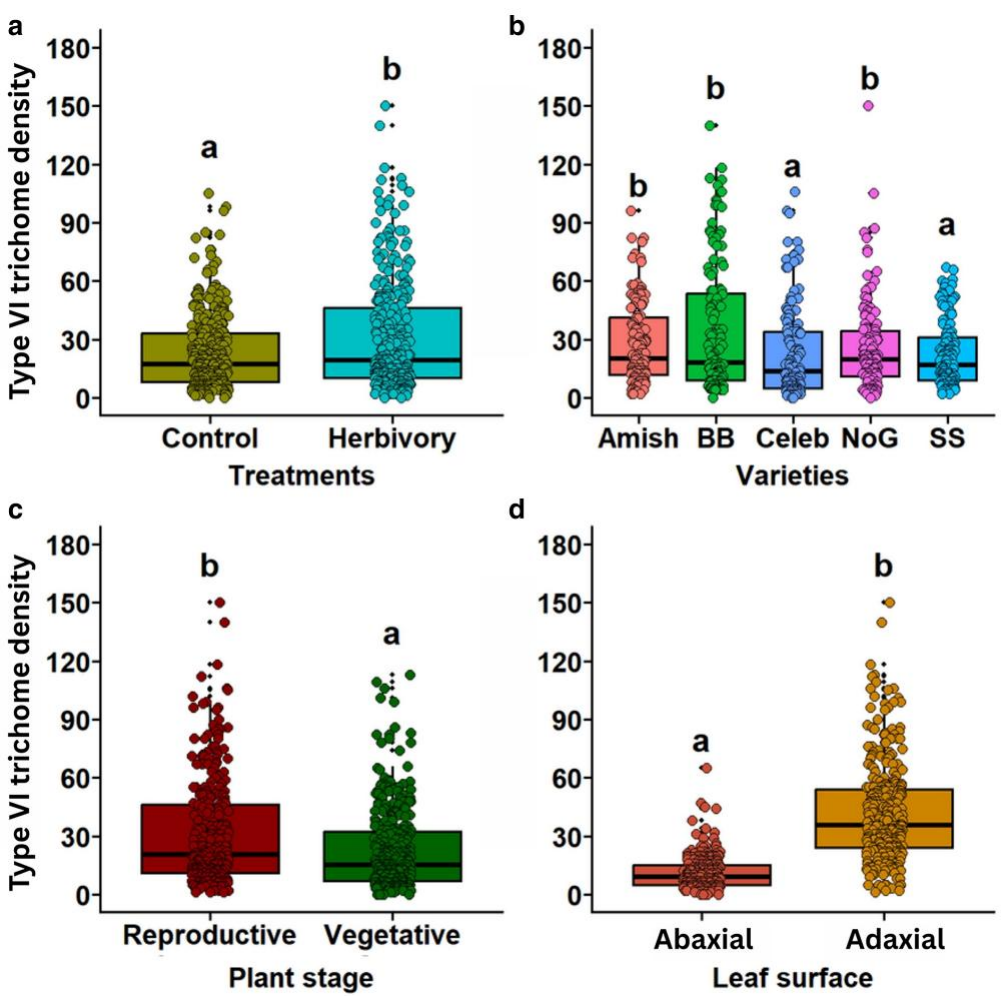
Density of Type VI trichomes on the surface of tomato leaf. Number of Type VI trichomes a) with and without herbivory, b) among tomato varieties, c) between plant stages, and d) between leaf surfaces. Varieties: Amish—Amish Paste Organic; BB—Big Beef (F1); Celeb—Celebrity Plus (F1); NoG—Nepal Organic; SS—Supersweet 100 (F1). The different alphabets indicate significant differences at the 5% level of significance.

Pooled mean trichome density showed significant variation in Type VI glandular trichome density among treatments (χ^2^ = 24.0, *P* < .001; Fig. 2a, Table S1), varieties (χ^2^ = 60.2, *P* < .001; Fig. 2b, Table S1), plant stages (χ^2^ = 66.7, *P* < .001; Fig. 2c, Table S1), and leaf surfaces (χ^2^ = 1059.3, *P* < .001; Fig. 2d, Table S1). Type VI trichome density was higher in herbivory-treated plants compared with control plants. Similarly, it was also higher on the adaxial surface than the abaxial surface of the tomato leaves and reproductive stage had higher density of Type VI trichomes when compared with the vegetative stage of plants. In terms of cultivars, Type VI trichome density was found to be significantly highest in Big Beef and Nepal Organic and lowest in Celebrity Plus and Super Sweet 100.

#### Interaction effect on Type VI glandular trichome

The interaction of treatment × variety (χ^2^ = 19.1, *P* < .001; Fig. S1a), variety × surface (χ^2^ = 90.2, *P* < .001; Fig. S1b), treatment × surface (χ^2^ = 14.8, *P* < .001; Fig. S1c), and variety × plant stage (χ^2^ = 68.8, *P* < .001; Fig. S1d) were found to have significant effects on the density of Type VI glandular trichomes (Table S1). The trichome density of Type VI trichomes was highest on the herbivory-treated Big Beef variety of tomato, but it was lowest on control treatment in Celebrity Plus. Two-way analysis revealed that Trichome VI density in Big Beef F1, Celebrity Plus F1, Supersweet 100 F1, and Amish Paste Organic were significantly higher for herbivory-treated plants than control plants, but the opposite was seen in the Nepal Organic variety (Fig. S1a). Similarly, the adaxial surface had more Type VI glandular trichome density in herbivory-treated plants as compared with control plants (Fig. S1c). For all varieties, trichome density was significantly higher on the adaxial than the abaxial surface of the tomato leaves (Fig. S1b). When comparing phenological stages across varieties, Nepal Organic exhibited the highest density of Type VI glandular trichomes during the reproductive stage, whereas Celebrity Plus F1 displayed the lowest density during the vegetative stage (Fig. S1d). The two-way interactions among treatment × plant stage and surface × plant stage; the three-way interaction among treatment × variety × surface, treatment × variety × plant stage, treatment × surface × plant stage, and variety × surface × plant stage, and the four-way interaction treatment × variety × surface × plant stage had non-significant effects on Type VI trichome density (Table S1).

#### Non-glandular trichomes

**Figure 3.**
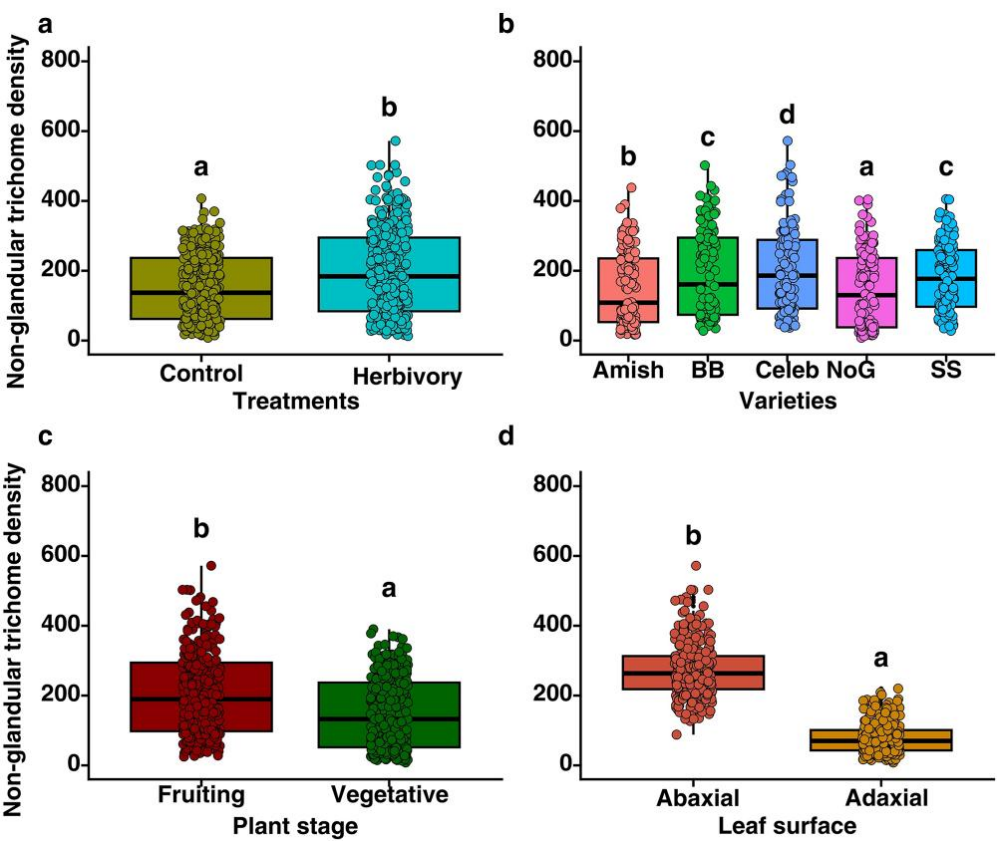
Density of NGTs on the surface of tomato leaf a) with and without herbivory, b) among tomato varieties, c) between plant stages, and d) between leaf surfaces. Varieties: Amish—Amish Paste Organic; BB—Big Beef (F1); Celeb—Celebrity Plus (F1); NoG—Nepal Organic; SS—Supersweet 100 (F1). The different alphabets indicate significant differences at the 5% level of significance.

Pooled mean trichome density showed significant variation in NGT density among treatments (χ^2^ = 272.0, *P* < .0001; Fig. 3a, Table S1), varieties (χ^2^ = 523.0, *P* < .0001; Fig. 3b), plant stages (χ^2^ = 701.0, *P* < .0001; Fig. 3c) and leaf surfaces (χ^2^ = 6837.7, *P* < .001; Fig. 3d). Among varieties, Celebrity Plus had the highest NGT density while it was lowest in Nepal Organic. Similarly, trichome density was significantly higher under herbivory treatments across all varieties compared with control. In terms of phenological stages, the reproductive stage had the highest NGTs, while across leaf surfaces, abaxial surface had highest NGT density.

#### Interaction effect on non-glandular trichomes

The interaction of treatment × variety (χ^2^ = 23.0, *P* < .001; Fig. S2a), variety × surface (χ^2^ = 240.0, *P* < .001; Fig. S2b), treatment × plant stage (χ^2^ = 62.8, *P* < .001; Fig. S2c), variety × plant stage (χ^2^ = 106.8, *P* < .001; Fig. S2d), and surface × plant stage (χ^2^ = 151.6, *P ≤* .001; Fig. S2e) were found to have significant effects on NGT density (Table S1). However, the interaction between treatment × leaf surface was non-significant (χ^2^ = 1.5, *P* = .22; Table S1). NGT density was highest on the leaf surface of the herbivory-treated Celebrity Plus variety, but it was lowest on control plants of the Nepal Organic variety (Fig. S2a). Likewise, the abaxial surface had more NGTs in Celebrity Plus while the lowest was observed on the adaxial surface of Nepal Organic tomato when considering the surface and variety interaction (Fig. S2b). Similarly, the interaction between the phenological stage and herbivory treatment showed that leaves from the reproductive stage under herbivory treatment had the highest NGTs. At the same time, the lowest was present in control treatments of vegetative stage leaves (Fig. S2c).

The interaction of treatment × variety × surface (χ^2^ = 48.0, *P* < .001; Fig. S3a) and treatment × variety × plant stage (χ^2^ = 30.3, *P* < .0001; Fig. S3b, Table S1) showed significant effects on the trichome density. Whereas interactions among treatment × surface × plant stage and variety × surface × plant stage were not significant (Table S1). Trichome density was highest on the abaxial surface of the herbivory-treated Celebrity Plus F1 variety, while it was lowest on the adaxial surface of leaves in control plants of Nepal Organic (Fig. S3a). Similarly, Celebrity Plus (F1) had more NGT density at the reproductive stage after herbivory treatment, whereas the lowest was observed at the vegetative stage in control plants of Nepal Organic tomato (Fig. S3b).

Overall interaction among four factors (herbivory, variety, surface, and phenological stage) (χ^2^ = 26.3, *P* < .001; Fig. S4, Table S1) revealed that herbivory-treated plants at reproductive stages have more trichome density on the abaxial surface of Celebrity Plus F1 which consistently had more NGT density in all interaction combinations (Fig. S4). Nepal organic and Amish Paste have the lowest density of trichome on the adaxial surface of the control plants at the vegetative stage (Fig. S4). These results show that NGT density in tomato plants are induced by herbivory damage. They may vary significantly across phenological stages and are highly dependent on genotypic characteristics.

#### Total leaf trichome density

**Figure 4.**
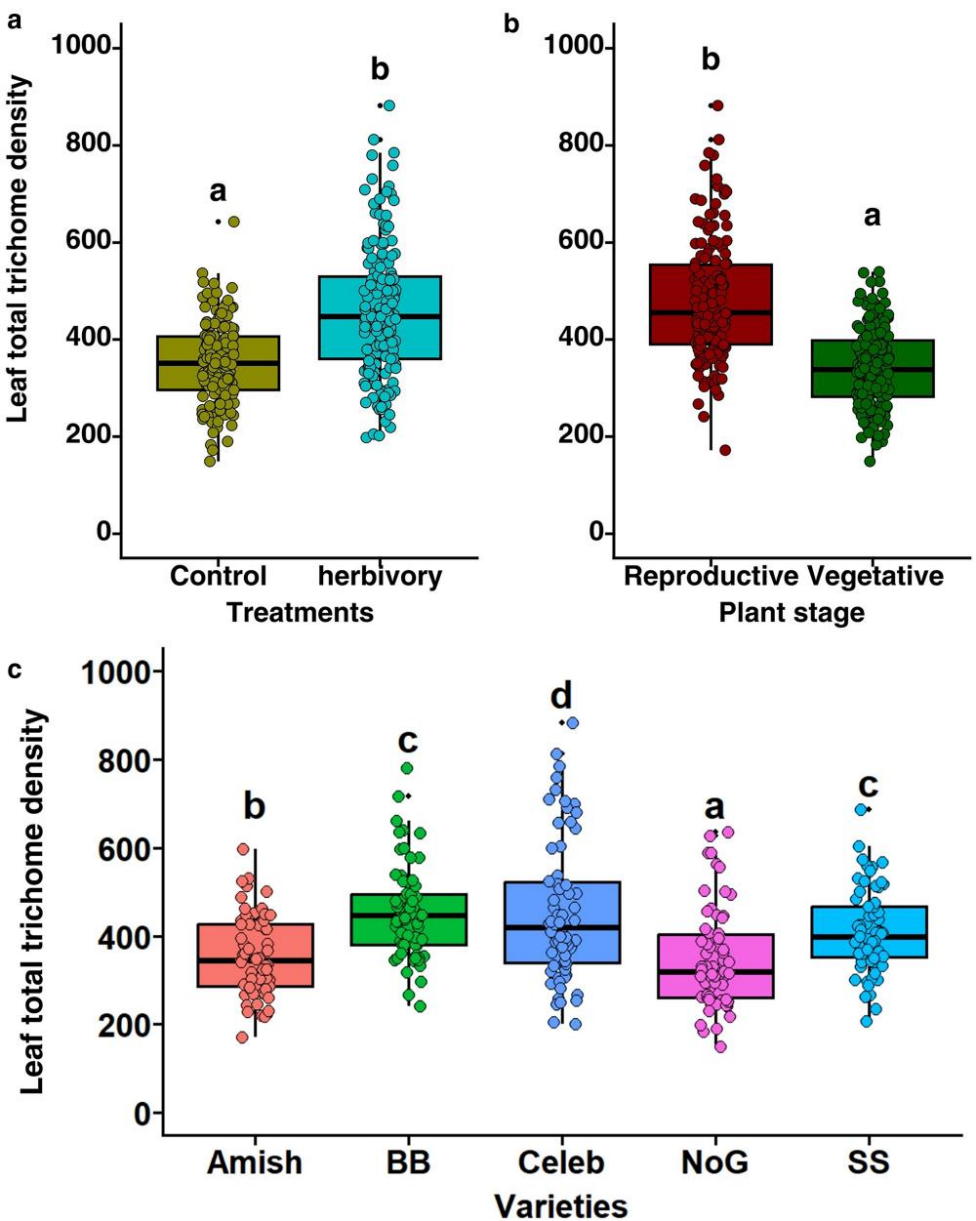
Leaf total trichome density a) with and without herbivory, b) between plant stages, and c) across tomato varieties. Varieties: Amish—Amish Paste Organic; BB—Big Beef (F1); Celeb—Celebrity Plus (F1); NoG—Nepal Organic; SS—Supersweet 100 (F1). The different alphabets indicate significant differences at the 5% level of significance.

To evaluate the total leaf trichome density, all trichome types were combined and aggregated across both the adaxial and abaxial leaf surfaces. Pooled mean total trichome density showed significant variation between herbivory treatments (χ^2^ = 222.9, *P* < .001; Fig. 4a), phenological stages (χ^2^ = 405.9, *P* < .001; Fig. 4b), and varieties (χ^2^ = 206.6, *P* < .001; Fig. 4c, Table S2). The total trichome density was found to be significantly induced under herbivory treatment by *S. exigua.* However, based on phenological stages, leaves collected from the reproductive stage had the highest total leaf trichome density compared with the vegetative stage. Moreover, across five different varieties, the total leaf trichome density was highest in Celebrity Plus variety, while the lowest was observed in the Nepal Organic tomato variety (Fig. 4c).

#### Interaction effect on total trichomes

**Figure 5.**
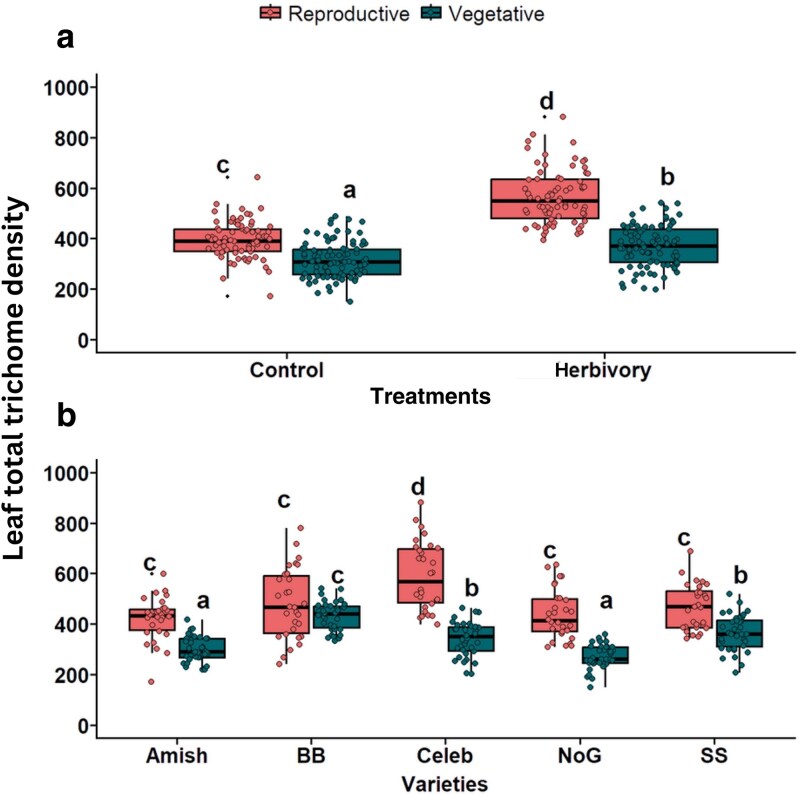
Leaf total trichome density under the a) interaction between treatments (control and herbivory) and plant stages (vegetative and reproductive), and b) interaction between varieties and plant stages. Varieties: Amish—Amish Paste Organic; BB—Big Beef F1; Celeb—Celebrity Plus F1; NoG—Nepal Organic; SS—Supersweet 100 F1. The different alphabets indicate significant differences at the 5% level of significance.

The interaction of treatment × plant stage (χ^2^ = 35.9, *P* < .001; Fig. 5a) and variety × plant stage (χ^2^ = 94.5, *P* < .001) on total trichome density were found to have significant effects on the total leaf trichomes (Fig. 5b). In contrast, treatment × variety has no significant impact on density of total trichome (Table S2; χ^2^ = 4.93, *P* = .29). Treatment × plant stage interaction revealed that the total trichome density on a leaf was highest in herbivory-treated plants of reproductive stages and lowest in control plants in their vegetative stages (Fig. 5a). Total trichome density was highest on the herbivory-treated Celebrity Plus variety of tomato, but it was lowest on the leaf surface of the control plant of Nepal Organic and Amish Paste (Fig. 5b). The three-way interaction of total trichome density among treatment × variety × plant stages was found to be non-significant (Table S2; χ^2^ = 7.4, *P* = .11).

### Time to initiate feeding experiment

**Figure 6.**
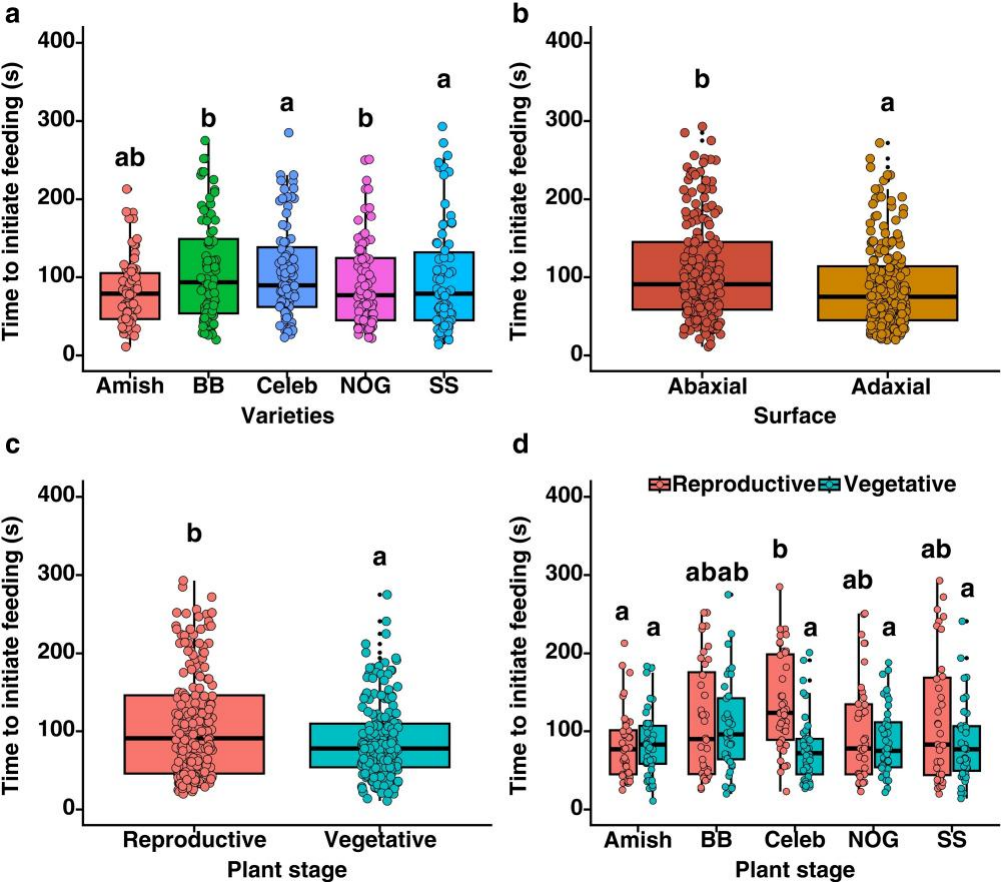
Time (seconds) taken by *S. exigua* first instar larvae to successfully start feeding initiation after being placed on the surface of five tomato varieties either on abaxial or adaxial surfaces with varying density of trichome numbers. Amish—Amish Paste Organic; BB—Big Beef F1; Celeb—Celebrity Plus F1; NoG—Nepal Organic; SS—Supersweet 100 F1. The different alphabets indicate significant differences at the 5% level of significance.

The initiation time for feeding by *S. exigua* varied significantly among different leaf varieties across both vegetative and reproductive stages. Across the varieties, the time taken to initiate feeding by *S*. *exigua* was the longest for Big Beef F1 and Nepal Organic (χ^2^ = 145.2, *P* = .02; Fig. 6a). On the abaxial surface of the leaves, *S. exigua* took the longest time to initiate feeding (χ^2^ = 141.1, *P* < .001; Fig. 6b). Likewise, *S. exigua* larvae took a longer time to initiate feeding on reproductive stage leaves than vegetative stage (χ^2^ = 137.5, *P* = .001; Fig. 6c). Two-way interaction of variety × plant stage revealed that leaves of the Celebrity Plus variety at the reproductive stage delayed the larva to initiate feeding (χ^2^ = 131.7, *P* = .01; Fig. 6d). Although the larvae were able to initiate feeding quickly in the vegetative stage, they were able to initiate feeding more quickly on Amish Paste Organic variety at both stages (Fig. 6b). At the reproductive stage, the mean total trichome from the leaf of the Celebrity Plus F1 variety was highest (699.73 ± 23.04), which may have delayed the time to initiate feeding in this variety, while time taken to initiate feeding was shortest on Nepal Organic and Amish Paste Organic at vegetative stage as total mean trichome was 286.83 ± 10.43 and 319.22 ± 10.92, respectively, when compared with reproductive stages, which may be the cause to quick feeding response shown by *S. exigua* larvae (Fig. 6b). The regression analysis showed that there was a strong positive correlation between time to initiate feeding and total trichomes in Celebrity Plus F1, Big Beef F1, and Nepal Organic varieties when examined under interactive effects of variety × surface and variety × stage (Figs S5 and S6). Furthermore, at the reproductive stage, there was a significant positive linear relationship between trichome density and time to initiate feeding by *S. exigua* when pooled across all varieties and leaf surfaces. This shows that leaf trichomes offer a substantial barrier to *S. exigua* larvae, which delays their feeding in tomato leaves, but varietal differences, leaf surface, and phenological stages drive this host–herbivore interaction via trichome.

## Discussion

In the present study, we quantified the density of Type VI glandular trichomes and NGTs on both leaf surfaces (adaxial and abaxial) and total leaf trichomes with and without herbivory by *S. exigua* at the vegetative and reproductive stages of five different tomato varieties. Through a comprehensive examination of trichomes, we show that leaf trichomes in tomatoes are highly variable and are strongly affected by varietal differences. In addition, leaf trichomes vary significantly across leaf surfaces and phenological stages, with the reproductive stage having denser trichomes than the vegetative stage. Our study also demonstrates that trichomes are readily induced upon herbivory damage by *S. exigua* and act as a substantial physical barrier against the larva by delaying their feeding. Overall, trichomes (both glandular and NG) could be traits of interest that can be of use in breeding resilient horticultural crops.

### Herbivory, phenological stage, leaf surface, and variety impact Type VI glandular trichomes

Tomato leaves comprise a wide range of trichomes including both glandular trichomes and NGTs. Glandular trichomes primarily provide plant defence through the production of chemical compound, whereas NGTs are basically responsible for physical defence against herbivory (Wagner et al. 2004, Glas et al. 2012, Karabourniotis et al. 2020). Among glandular trichomes observed in our study, Type VI glandular trichomes were found most abundantly (Fig. 1), and similar findings have been reported in many other cultivated *So. lycopersicum* species (Maluf et al. 2007, McDowell et al. 2011, Watts and Kariyat 2021b).

Based on the analysis of pooled data in this study, results show that Type VI glandular trichomes were significantly induced under herbivory treatment. This could be due to the induction of JA and SA upon herbivory as it has been well understood that damage by herbivores can induce JA-SA-mediated signalling cascades in the plants (Gautam and Kariyat 2025b). The induction of JA or SA in turn can lead to an increase in the production of Type VI trichomes and significantly induce polyphenol oxidase activity (Chen et al. 2018, Yuan et al. 2021, Zheng et al. 2025). Along with the induction of glandular trichomes, protease inhibitors and terpene induction have been found to occur in tomatoes when herbivores walk on the leaves and rupture the glandular trichomes. Surprisingly, the interaction between the phenological stages and herbivory treatment was non-significant on Type VI glandular trichomes. This showed that the effects observed in this study were independent of the stages of the plants, and the trichomes were similarly induced at both vegetative and reproductive stages by *S. exigua* feeding. However, our study did not delve into the exudates from the glandular trichomes upon herbiovry by *S. exigua* and the metabolites involved were not analysed. Understanding the secondary metabolites associated with glandular trichomes and quantifying phytohormonal regulation can aid in enhancing our knowledge about glandular trichome-mediated defences in tomato and other crops against herbivores.

Besides herbivory, the Type VI glandular trichome was found to be significantly different across the phenological stages when analysed independently. We observed that reproductive stage had greater Type VI glandular trichome and NGT density than the vegetative stage. Similar to our findings, the density of Type VI trichomes has been found to increase significantly in *L*ycopersicon *hirsutum* as the plant aged, from two months old (plants with flowers and small fruits) to four months old plants with flowers and mature fruits (Leite et al. 2001). Leite et al. (2001) reported an increase in trichome density with plant age, and it was associated with the increment in the levels of tridecan-2-one compounds in *L. hirsutum* glandular trichomes. It seems like with progressing phenological stage of tomatoes, the newly formed leaves produce more Type VI glandular trichomes. We speculate that the plants’ goal in maintaining the same level of defence via Type VI defence is to minimize damage to reproductive structures of the plants from potential herbivory incidence. However, some studies have found no difference in trichome production between vegetative and reproductive stages of tomato (Mymko and Avila-Sakar 2019). The variation in glandular trichomes across phenological stages is still a debatable topic since there is hypothesized to be a cost or a trade-off associated with maintaining the same level of defence and production of reproductive structures (Strauss and Agrawal 1999). In our study, we did not quantify the yield and fitness of the tomato varieties and thus were unable to truly determine the extent of defence fitness trade-off. We also expected to see differences in glandular trichomes across the leaf surfaces across the vegetative and reproductive stages. However, the interaction between the leaf surface and plant stage was non-significant.

Across the leaf surface, Type VI glandular trichome density was found to be significantly more abundant on the adaxial than on the abaxial surface. Both the independent effects of surface (Fig. 2) and the interaction effects of leaf surface with herbivory treatment and varieties (Fig. S1) support this finding. Furthermore, this result is consistent with Bergau et al. (2015), who reported that the developmental progression of Type VI glandular trichomes was denser on the adaxial surface of both *Solanum habrochaites* (a wild variety) and cultivated tomato. Additionally, Bosabalidis and Skoula (1998) observed that more glandular trichomes were present on the upper surface compared with the lower surface of *Origanum* × *intercedens*, which they suggested might be associated with UV radiation and insect attack. As it seems, glandular trichomes can be highly affected by the level of light they receive and the incidence of herbivory. For example, Escobar-Bravo et al. (2018) reported that high photosynthetically active radiation (PAR) can increase Type VI trichome density on adaxial leaf sides of two varieties of tomato leaves. Although such variations can differ according to the growing conditions, and the varieties, 16 out of 21 genotypes were found to have higher glandular trichome density (number of trichomes/0.5 mm^2^) on the adaxial surface of the tomato leaves (Sridhar et al. 2019). Although a consensus can be established that glandular trichomes are higher in adaxial surface, we also have to take into account the numerous variations present in different varieties that are bred to possess unique traits against different stressors.

This study used five commercially grown varieties of tomato and we found that Big Beef and Nepal Organic had significantly greatest density of Type VI trichome, whereas Super Sweet and Celebrity Plus had the lowest. Glandular trichomes on the abaxial and adaxial surfaces can vary significantly among the varieties (Nord et al. 2020), and density could range from 1 to 82 on the abaxial and 9–90 on the adaxial surface in a 0.8 mm^2^ leaf area as observed in this study. Similar to the findings of this study, certain tomato cultivars like LA2695, LA1578, and LA1718 have been found to possess more Type VI trichomes (Kortbeek et al. 2021). Additionally, we also found that the varieties also showed interaction effects with the herbivory treatment, leaf surface and plant stages. This indicates that although independent effects were observed for all other factors, variety still plays an important role in determining the presence and induction of Type VI trichomes in tomato plants. Differential densities of Type VI trichomes among different tomato varieties have been described by Kaur et al. (2023), and several past studies also suggest that varieties with different glandular trichome densities in tomatoes can potentially offer greater resistance to herbivory (Neiva et al. 2013, da Silva et al. 2018). Such immense diversity in the presence of glandular trichomes in different varieities gives us an opportunity to select the desired trait of glandular trichomes that could be inherited from backcrossing the domestic varieties to wild varieties of tomato (de Resende et al. 2022) for developing resilient cultivars.

### Herbivory, phenological stage, leaf surface, and variety induce non-glandular trichomes

Similar to the Type VI glandular trichomes, NGTs were also significantly induced by *S. exigua* damage. This was within our expectations as NGTs have been widely studied and found to be readily induced as a coping mechanism against biotic and abiotic stress in tomatoes and many other crops (Traw and Dawson 2002, Gonzáles et al. 2008). However, compared with glandular trichomes, NGTs were more abundant in our study which is most likely due to the glandular trichomes’ requirement for an expensive investment. This inference is also supported by the images obtained under SEM which clearly showed the overwhelming presence of NGTs in tomato leaves compared with glandular trichomes. While glandular trichomes provide defences via production of secondary metabolites and chemical compounds that deter herbivores by both antixenosis and antibiosis, NGTs primarily confer physical defences to the plants (Sridhar et al. 2019, Riahi et al. 2023). Clearly, both glandular trichome and NGT have critical role to play in the ongoing battle between the hosts and the herbivores. However, like glandular trichome and NGT are also driven by several factors such as phenological stages, and leaf surface which vary significantly across different varieties.

In terms of phenological stages, the results show that reproductive stages had greater NGT density like that of Type VI glandular trichomes. However, a contrasting observation to our findings has been reported wherein the NGT density remained the same between the pre-blossom and blossom stages of tomato (Mulatu et al. 2006). This could be due to the effects of other factors, such as variety, leaf surface, herbivory treatment, and phenological stages, as all these factors have been found to interact and influence the production of NGT density. Compared with Type VI glandular trichome density, although we expected them to be induced similarly, the interactive effects of herbivory with plant stage were significant for the density of NGTs but non-significant for Type VI glandular trichomes. This could be mainly due to the highly dynamic and cost-effective nature of NGTs owing to their focus on physical defence compared with the glandular trichomes which require huge investment for the induction of glandular trichomes armed with chemical defences (Gonzáles et al. 2008). These inferences indicate a potential trade-off between the production of glandular and NGTs in tomatoes which is only strengthened when compared across the leaf surface.

Our results reveal that NGT density was significantly higher in abaxial surface of the leaves, whereas glandular trichome density was higher in the adaxial surface. The effect is prevalent even under the interactive effects of varieties and leaf surface, and phenological stage and leaf surface. Most importantly, the interactive effects of herbivory and leaf surface showed that NGT density was significantly higher on abaxial surface which is in contrast to higher Type VI glandular trichome density found on adaxial surface. According to our results, NGTs were denser on the abaxial surface by two- to three-folds. A possible explanation for higher NGTs on abaxial surface could be due to greater herbivore activity on abaxial compared with adaxial surface. For example, most lepidopteran insect herbivores deposit their eggs on abaxial surface (Azidah and Sofian-Azirun 2006, Hilker and Meiners 2006), probably to avoid biotic and abiotic stresses, which could have led the plants to possess more NGTs on the abaxial surface. Moreover, previous studies suggested that glandular trichomes are highly influenced by the light conditions, particularly UV radiation and PAR compared with NGTs (Escobar-Bravo et al. 2018). Our results show a clear distinction between the induction of glandular trichome and NGT across the leaf surfaces of tomatoes. These results, however, are largely influenced by the inherent varietal traits.

In the current experiment, Celebrity Plus F1 variety exhibited the highest density of NGTs while NoG had the lowest which is in contrast to the Type VI glandular trichome density. Past studies have shown that there is a strong genetic basis for trichome density as well as type of trichomes in tomatoes (Paudel et al. 2019, de Souza Marinke et al. 2022, Zannou et al. 2025) conferring them abilities to withstand biotic and abiotic stressors. For example, varieties which are mostly abundant in terms of NGTs usually have a better water storage capacity and perform better under water stress (Zhang et al. 2020). Furthermore, we observed that varieties also interacted with herbivory treatment, and leaf surface, phenological stages to affect the NGT density. These results strongly suggest that while multi-fold factors determine the density and the type of trichomes, varietal effects play a significant role and should be considered when studying for the effects of different treatments on tomato leaf trichomes.

### Effects on total trichomes

Our results indicated that the total trichomes (including all types of trichomes across both surfaces) were significantly different based on herbivory treatment, phenological stages, and varieties. As expected, the total trichome density was also significantly induced by the herbivory treatment, and we found greater trichome density in leaves from the reproductive stage compared with the vegetative stage, as observed in the case of Type VI glandular trichome and NGTs. Moreover, Celebrity Plus variety had the greatest total trichomes, while Nepal Organic had the least, which is consistent with the results obtained for both trichome types. The varieties were found to interact with phenological stages, and certain tomato varieties, Big Beef, Celebrity Plus, and Nepal Organic demonstrated higher trichome density during the reproductive stage. During the reproductive stage, plants often face increased vulnerability due to herbivory, which can impact their resource allocation. It is suggested that plants may invest more in defensive structures, such as trichomes, to respond to stressors. Plants usually tend to have poor fitness when more resources are allocated towards trichome production, as the plant prioritizes defence over reproduction. For instance, (Kariyat et al. 2013) reported an increase in trichome density on adaxial and abaxial leaf surfaces of both inbred and outbred progenies of *Solanum carolinense* damaged by *Manduca sexta* larvae, which compromised flower production and led to fewer flowers. However, since we did not record the number of flowers and fruit set in our study, it is difficult to come to the conclusion with this hypothesis. Therefore, future experiments should explore the pattern of trichome production along with reproductive traits such as flowering and fruit setting to understand the underlying trade-off mechanisms in tomato varieties.

Our comprehensive examination of total trichomes and different types of trichomes reveal that interactions between herbivory damage, variety, leaf surface, and phenological stages are crucial for understanding the holistic nature of trichome development in tomatoes. Furthermore, our results present a holistic approach to understanding the isolated as well as interactive effects of several factors on trichomes which adds to the novel insights inferred from this study. The results underscore the combined effects of genetic factors, growth stage, herbivory treatments, and leaf surface properties of tomato plants on leaf trichome density.

### Tomato leaf trichomes delay feeding by *Spodoptera exigua*

The time taken to initiate feeding by first instar larvae of *S. exigua* differed significantly among the varieties and leaf surfaces. The larva took significantly more time to begin feeding on the abaxial surface than the adaxial surface, which is in line with the findings from Watts and Kariyat (2021a), specifically for early instar larvae. Delayed feeding on the abaxial surface can be attributed to the presence of high density of NGTs (Kariyat et al. 2018), which are responsible for impeding larvae movement and damaging insect gut upon ingestion (Kariyat et al. 2017b). It has also been observed that such foliage-feeding insect herbivores wander for a longer duration on leaf surfaces to find suitable spots or to remove the hairy trichomes before actual feeding (Shelomi et al. 2010). Two-way factorial analysis showed that the Celebrity Plus variety at the reproductive stage slowed down the larva and delayed feeding, which was at par with Supersweet 100 F1 variety. In contrast, the larvae were significantly faster at initiating feeding on the Amish Paste Organic variety. Since Celebrity Plus variety had denser trichomes, it is clear that denser trichomes aid the plants in restricting larval movements and delaying feeding, which allows the plants to mount further defences against such insect herbivores. For instance, Zannou et al. (2025) reported the longer time taken for larvae to settle on leaflets with higher trichome density. Similarly, Mulatu et al. (2006) also showed significant interaction of plant stage, cultivar, and leaf surface on the settling of potato tuber moth on the leaf of a tomato. Regression analysis showed that the trichome density had a linear relationship with the time to initiate feeding for variety × leaf surface (Fig. S5), variety × phenological stages (Fig. S6), and variety × phenological stages (Fig. S7). However, these effects could also be due to the volatile organic compounds (VOCs) as they have critical role in host–herbivore interactions (Lin et al. 2022, Debnath et al. 2024, Gandham et al. 2024). Hence, the effects of VOCs and other secondary metabolites should be considered while examining the effects of trichomes on herbivore activities. Nevertheless, the results from the current study demonstrate that trichomes are a crucial physical defence structure in tomato plants, and their density and types are subjected to changes according to the varieties, leaf surface, and phenological stages of the plants. Therefore, trichomes, being an excellent defence structure, should be given priority in varietal developmental programmes for developing resilient agricultural crops.

## Conclusion

In this study, we showed that herbivory induces glandular trichome and NGT in tomato which vary across varieties, their phenological stages, and leaf surfaces. Highest trichome density for both glandular trichome and NGT was observed in reproductive stage, whereas across the leaf surface opposing results were found according to the trichome type. Glandular trichomes were denser on the adaxial surface, whereas NGTs were abundant on the abaxial surface. Overall, our results strongly demonstrate that a combination of factors such as herbivory treatment, leaf surface, phenological stages, and variety significantly influences trichome density. Notably, for glandular trichomes, significant interactions were observed between variety × treatment, variety × surface, treatment × surface, and variety × plant stage. Similarly, for NGTs, significant two-way interactions were observed between variety × treatment, variety × surface, treatment × plant stage, variety × plant stage, and leaf surface × plant stage. Moreover, the three-way (treatment × variety × surface and treatment × variety × plant stage) and the four-way significant interaction (treatment × variety × surface × plant stage) further highlighted that regulation of the trichome is a complex phenomenon and involves multiple factors, including genotype × environmental effects. This highlights the multifaceted plant defence strategies related to the upregulation of trichome production that have both intrinsic (genetic) as well as external factors that contribute to it. These multifactor relationships from our study provide valuable insights into plant defence mechanisms and offer a foundation for breeding or managing crop varieties, which may help breed varieties that are resistant to herbivory.

## Supplementary Material

plaf057_Supplementary_Data

## Data Availability

The raw data and the statistical analysis are available in Supplementary materials S1 and S2.

## References

[plaf057-B1] Agrawal A . Transgenerational consequences of plant responses to herbivory: an adaptive maternal effect? Am Nat 2001;157:555–69. 10.1086/319932 18707262

[plaf057-B2] Ayala J, Vasquez A, Balakrishnan D et al Effects of fast and slow-wilting soybean genotypes on fall armyworm (*Spodoptera frugiperda*) growth and development. Commun Integr Biol 2024;17:2354421. 10.1080/19420889.2024.2354421 38778870 PMC11110702

[plaf057-B3] Azidah AA, Sofian-Azirun M. Some aspects on oviposition behaviour of *Spodoptera exigua* (Hübner) (Lepidoptera: Noctuidae). J Entomol 2006;3:261–6. 10.3923/je.2006.261.266 17201979

[plaf057-B4] Balakrishnan D, Srivastava V, Kariyat R. Rice sucrose non-fermenting related protein kinase (SnRK1) has a limited role in defense against fall armyworm (*Spodoptera frugiperda*). Plant Stress 2024;14:100667. 10.1016/j.stress.2024.100667

[plaf057-B5] Bergau N, Bennewitz S, Syrowatka F et al The development of type VI glandular trichomes in the cultivated tomato *Solanum lycopersicum* and a related wild species *S. habrochaites*. BMC Plant Biol 2015;15:1–15. 10.1186/s12870-015-0678-z 26654876 PMC4676884

[plaf057-B6] Bosabalidis AM, Skoula M. A comparative study of the glandular trichomes on the upper and lower leaf surfaces of *origanum* × *intercedens* rech. J Essent Oil Res 1998;10:277–86. 10.1080/10412905.1998.9700900

[plaf057-B7] Boughton AJ, Hoover K, Felton GW. Methyl jasmonate application induces increased densities of glandular trichomes on tomato, Lycopersicon esculentum. Journal of Chemical Ecology 2005;31:2211–6. 10.1007/s10886-005-6228-7 16132222

[plaf057-B8] Channarayappa C, Shivashankar G, Muniyappa V et al Resistance of Lycopersicon species to bemisia tabaci, a tomato leaf curl virus vector. Can J Bot 1992;70:2184–92. 10.1139/b92-270

[plaf057-B9] Chen G, Klinkhamer PGL, Escobar-Bravo R et al Type VI glandular trichome density and their derived volatiles are differently induced by jasmonic acid in developing and fully developed tomato leaves: implications for thrips resistance. Plant Sci 2018;276:87–98. 10.1016/j.plantsci.2018.08.007 30348331

[plaf057-B10] Chowdhury R, Grafe TU, Metali F et al Arms race of physical defences: hooked trichomes of Macaranga ant-plants kill lycaenid caterpillars, but one specialist has a counter-defence. Biol Lett 2025;21:1–6. 10.1098/rsbl.2025.0005 PMC12142895 40476537

[plaf057-B11] da Silva AA, Carvalho R de C, Andrade MC et al Glandular trichomes that mediate resistance to green peach aphid in tomato genotypes from the cross between *S. galapagense* and *S. lycopersicum*. Acta Sci Agron 2018;41:e42704–8. 10.4025/actasciagron.v41i1.42704

[plaf057-B12] Debnath R, George J, Gautam M et al Ecological interactions, host plant defenses, and control strategies in managing soybean looper, *Chrysodeixis includens* (Lepidoptera: Noctuidae). Front Insect Sci 2024;4:1480940. 10.3389/finsc.2024.1480940 39726917 PMC11669695

[plaf057-B13] de Resende JTV, Dias DM, Erpen-Dalla Corte L et al The introgression of resistance to *Tuta absoluta* in tomato based on glandular trichomes. Arthropod Plant Interact 2022;16:87–99. 10.1007/s11829-021-09873-x

[plaf057-B14] de Souza Marinke L, de Resende JTV, Hata FT et al Selection of tomato genotypes with high resistance to *Tetranychus evansi* mediated by glandular trichomes. Phytoparasitica 2022;50:629–43. 10.1007/s12600-022-00984-6

[plaf057-B15] Eigenbrode SD, Trumble JT. Antibiosis to beet armyworm (*Spodoptera exigua*) in Lycopersicon accessions. HortScience 1993;28:932–4. 10.21273/HORTSCI.28.9.932

[plaf057-B16] Eigenbrode SD, Trumble JT, White KK. Trichome exudates and resistance to beet armyworm (lepidoptera: noctuidae) in *Lycopersicon hirsutum* f. *typicum* accessions. Environ Entomol 1996;25:90–5. 10.1093/ee/25.1.90

[plaf057-B17] Escobar-Bravo R, Ruijgrok J, Kim HK et al Light intensity-mediated induction of trichome-associated allelochemicals increases resistance against thrips in tomato. Plant Cell Physiol 2018;59:2462–75. 10.1093/pcp/pcy166 30124946 PMC6290487

[plaf057-B18] Gairola S, Naidoo Y, Bhatt A et al An investigation of the foliar trichomes of *Tetradenia riparia* (Hochst.) Codd [Lamiaceae]: an important medicinal plant of Southern Africa. Flora Morphol Distrib Funct Ecol Plants 2009;204:325–30. 10.1016/j.flora.2008.04.002

[plaf057-B19] Gandham K, Gautam M, George J et al Muffled olfactory and sensory cues from the reproductive stage soybean selectively reduce oviposition of a major polyphagous herbivore, fall armyworm (*Spodoptera frugiperda*). Pest Manag Sci 2024. 10.1002/ps.8600 PMC12539390 39710863

[plaf057-B20] Gautam M, Kariyat R. Drought and herbivory have selective transgenerational effects on soybean eco—physiology, defence and fitness. Plant Cell Environ 2025a. 10.1111/pce.70067 40653760

[plaf057-B21] Gautam M, Kariyat R. Drought and herbivory drive physiological and phytohormonal changes in soybean (*Glycine max* Merril): insights from a meta-analysis. Plant Cell Environ 2025b. 10.1111/pce.15558 40241323

[plaf057-B22] Glas JJ, Schimmel BCJ, Alba JM et al Plant glandular trichomes as targets for breeding or engineering of resistance to herbivores. Int J Mol Sci 2012;13:17077–103. 10.3390/ijms131217077 23235331 PMC3546740

[plaf057-B23] Gonzáles WL, Negritto MA, Suárez LH et al Induction of glandular and non-glandular trichomes by damage in leaves of *Madia sativa* under contrasting water regimes. Acta Oecol 2008;33:128–32. 10.1016/j.actao.2007.10.004

[plaf057-B24] Gyawali P, Hwang S, Sotelo-cardona P et al Elucidating the fitness of a dead-End trap crop strategy against the tomato fruitworm, *Helicoverpa armigera*. Insects 2021;12:506. 10.3390/insects12060506 34072729 PMC8227471

[plaf057-B25] Hagenbucher S, Olson DM, Ruberson JR et al Resistance mechanisms against arthropod herbivores in cotton and their interactions with natural enemies. CRC Crit Rev Plant Sci 2013;32:458–82. 10.1080/07352689.2013.809293

[plaf057-B26] Hilker M, Meiners T. Early herbivore alert: insect eggs induce plant defense. J Chem Ecol 2006;32:1379–97. 10.1007/s10886-006-9057-4 16718566

[plaf057-B27] Karabourniotis G, Liakopoulos G, Nikolopoulos D et al Protective and defensive roles of non-glandular trichomes against multiple stresses: structure–function coordination. J For Res (Harbin) 2020;31:1–12. 10.1007/s11676-019-01034-4

[plaf057-B28] Kariyat RR, Balogh CM, Moraski RP et al Constitutive and herbivore-induced structural defenses are compromised by inbreeding in *Solanum carolinense* (Solanaceae). Am J Bot 2013;100:1014–21. 10.3732/ajb.1200612 23545253

[plaf057-B29] Kariyat RR, Hardison SB, De Moraes CM et al Plant spines deter herbivory by restricting caterpillar movement. Biol Lett 2017a;13:20170176. 10.1098/rsbl.2017.0176 28490447 PMC5454246

[plaf057-B30] Kariyat RR, Hardison SB, Ryan AB et al Leaf trichomes affect caterpillar feeding in an instar-specific manner. Commun Integr Biol 2018;11:1–6. 10.1080/19420889.2018.1486653 PMC6132425 30214672

[plaf057-B31] Kariyat RR, Smith JD, Stephenson AG et al Non-glandular trichomes of *Solanum carolinense* deter feeding by *Manduca sexta* caterpillars and cause damage to the gut peritrophic matrix. Proc R Soc Lond B Biol Sci 2017b;284:20162323. 10.1098/rspb.2016.2323 PMC5326521 28228510

[plaf057-B32] Kaur J, Kariyat R. Role of trichomes in plant stress biology. In: Núñez-Farfán J, Valverde P (eds.) Evolutionary Ecology of Plant-Herbivore Interaction. Cham: Springer, 2020, pp. 15–35. 10.1007/978-3-030-46012-9_2

[plaf057-B33] Kaur I, Kariyat R. Trichomes mediate plant–herbivore interactions in two Cucurbitaceae species through pre- and post-ingestive ways. J Pest Sci (2004) 2023;96:1077–89. 10.1007/s10340-023-01611-x 37168103 PMC10047472

[plaf057-B34] Kaur S, Khanal N, Dearth R et al Morphological characterization of intraspecific variation for trichome traits in tomato (*Solanum lycopersicum*). Bot Stud 2023;64:7. 10.1186/s40529-023-00370-3 36988701 PMC10060485

[plaf057-B35] Khederi SJ, Khanjani M, Hosseini MA et al Role of different trichome style in the resistance of *Lycopersicon hirsutum* genotypes to *Tuta absoluta* (Meyrick) (Lepidoptera: Gelechiidae). Ecol Montenegrina 2014;1:55–63. 10.37828/em.2014.1.8

[plaf057-B36] Kortbeek RWJ, Galland MD, Muras A et al Natural variation in wild tomato trichomes; selecting metabolites that contribute to insect resistance using a random forest approach. BMC Plant Biol 2021;21:1–19. 10.1186/s12870-021-03070-x 34215189 PMC8252294

[plaf057-B37] Kwon M, Cho HM, Ahn YJ. Relationship between feeding damage by Beet armyworm, *Spodoptera exigua* (Lepidoptera: Noctuidae) and leaf trichome density of potato. J Asia-Pacific Entomol 2006;9:361–367.

[plaf057-B38] Lalitha Y, Ballal CR. A rearing protocol for *Spodoptera exigua*. Indian J Plant Protect 2015;43:521–3.

[plaf057-B39] Leite GLD, Picanço M, Guedes RNC et al Role of plant age in the resistance of *Lycopersicon hirsutum* f. *glabratum* to the tomato leafminer *Tuta absoluta* (Lepidoptera: Gelechiidae). Sci Horticult 2001;89:103–13. 10.1016/S0304-4238(00)00224-7

[plaf057-B40] Lin PA, Chen Y, Ponce G et al Stomata-mediated interactions between plants, herbivores, and the environment. Trends Plant Sci 2022;27:287–300. 10.1016/j.tplants.2021.08.017 34580024

[plaf057-B41] Maluf WR, Inoue IF, Ferreira RDPD et al Higher glandular trichome density in tomato leaflets and repellence to spider mites. Pesqui Agropecu Bras 2007;42:1227–35. 10.1590/S0100-204X2007000900003

[plaf057-B42] McDowell ET, Kapteyn J, Schmidt A et al Comparative functional genomic analysis of solanum glandular trichome types. Plant Physiol 2011;155:524–39. 10.1104/pp.110.167114 21098679 PMC3075747

[plaf057-B43] Mitchell C, Brennan RM, Graham J et al Plant defense against herbivorous pests: exploiting resistance and tolerance traits for sustainable crop protection. Front Plant Sci 2016;7:1–8. 10.3389/fpls.2016.01132 27524994 PMC4965446

[plaf057-B44] Mulatu B, Applebaum SW, Coll M. Effect of tomato leaf traits on the potato tuber moth and its predominant larval parasitoid: a mechanism for enemy-free space. Biol Control 2006;37:231–6. 10.1016/j.biocontrol.2005.12.007

[plaf057-B45] Mymko D, Avila-Sakar G. The influence of leaf ontogenetic stage and plant reproductive phenology on trichome density and constitutive resistance in six tomato varieties. Arthropod Plant Interact 2019;13:797–803. 10.1007/s11829-019-09690-3

[plaf057-B46] Neiva IP, de Andrade Júnior VC, Maluf WR et al Papel de aleloquímicos e densidade de tricomas na resistência de tomateiro à mosca-branca. Ciencia Agrotecnol 2013;37:61–7. 10.1590/S1413-70542013000100007

[plaf057-B47] Nord R, Cortez-Madrigal H, Rodríguez-Guzmán E et al Grafting wild tomato genotypes and Mexican landraces increases trichome density and resistance against pests. Southwest Entomol 2020;45:649–62. 10.3958/059.045.0308

[plaf057-B48] Ochoa-López S, Villamil N, Zedillo-Avelleyra P et al Plant defence as a complex and changing phenotype throughout ontogeny. Ann Bot 2015;116:797–806. 10.1093/aob/mcv113 26220657 PMC4590325

[plaf057-B49] Oney MA, Bingham RA. Effects of simulated and natural herbivory on tomato (*Solanum lycopersicum* var. *esculentum*) leaf trichomes. Bios 2014;85:192–8. 10.1893/0005-3155-85.4.192

[plaf057-B50] Paudel S, Lin PA, Foolad MR et al Induced plant defenses against herbivory in cultivated and wild tomato. J Chem Ecol 2019;45:693–707. 10.1007/s10886-019-01090-4 31367970

[plaf057-B51] R Core Team . R: A Language and Environment for Statistical Computing. 2023.

[plaf057-B52] Riahi C, Urbaneja A, Fernández-Muñoz R et al Induction of glandular trichomes to control *Bemisia tabaci* in tomato crops: modulation by the natural enemy *Nesidiocoris tenuis*. Phytopathology 2023;113:1677–85. 10.1094/PHYTO-11-22-0440-V 36998120

[plaf057-B53] Shelomi M, Perkins LE, Cribb BW et al Effects of leaf surfaces on first-instar *Helicoverpa armigera* (Hübner) (Lepidoptera: Noctuidae) behaviour. Aust J Entomol 2010;49:289–95. 10.1111/j.1440-6055.2010.00766.x

[plaf057-B54] Simmons AT, Gurr GM. Trichomes of Lycopersicon species and their hybrids: effects on pests and natural enemies. Agric For Entomol 2005;7:265–76. 10.1111/j.1461-9555.2005.00271.x

[plaf057-B55] Sridhar V, Sadashiva AT, Keshava Rao V et al Trichome and biochemical basis of resistance against *Tuta absoluta* in tomato genotypes. Plant Genetic Resources: Characterization and Utilization 2019;17:301–5. 10.1017/S147926211800062X

[plaf057-B56] Strauss SY, Agrawal AA. The ecology and evolution of plant tolerance to herbivory. Trends Ecol Evol 1999;14:179–85. 10.1016/S0169-5347(98)01576-6 10322530

[plaf057-B57] Tian D, Tooker J, Peiffer M et al Role of trichomes in defense against herbivores: comparison of herbivore response to woolly and hairless trichome mutants in tomato (*Solanum lycopersicum*). Planta 2012;236:1053–66. 10.1007/s00425-012-1651-9 22552638

[plaf057-B58] Traw MB, Dawson TE. Differential induction of trichomes by three herbivores of black mustard. Oecologia 2002;131:526–32. 10.1007/s00442-002-0924-6 28547547

[plaf057-B59] Voigt D, Gorb S. Locomotion in a sticky terrain. Arthropod Plant Interact 2010;4:69–79. 10.1007/s11829-010-9088-1

[plaf057-B60] Wagner GJ, Wang E, Shepherd RW. New approaches for studying and exploiting an old protuberance, the plant trichome. Ann Bot 2004;93:3–11. 10.1093/aob/mch011 14678941 PMC4242265

[plaf057-B61] War AR, Paulraj MG, Ahmad T et al Mechanisms of plant defense against insect herbivores. Plant Signal Behav 2012;7:1306–20. 10.4161/psb.21663 22895106 PMC3493419

[plaf057-B62] Watts S, Kariyat R. Picking sides: feeding on the abaxial leaf surface is costly for caterpillars. Planta 2021a;253:1–6. 10.1007/s00425-021-03592-6 33661399

[plaf057-B63] Watts S, Kariyat R. Morphological characterization of trichomes shows enormous variation in shape, density and dimensions across the leaves of 14 Solanum species. AoB Plants 2021b;13:plab071. 10.1093/aobpla/plab071 34917310 PMC8670628

[plaf057-B64] Wilkens RT, Shea GO, Halbreich S et al Resource availability and the trichome defenses of tomato plants. Oecologia 1996;106:181–91. 10.1007/BF00328597 28307642

[plaf057-B65] Yuan Y, Xu X, Luo Y et al R2r3 MYB-dependent auxin signalling regulates trichome formation, and increased trichome density confers spider mite tolerance on tomato. Plant Biotechnol J 2021;19:138–52. 10.1111/pbi.13448 32654333 PMC7769234

[plaf057-B66] Zannou AJ, Romeis J, Collatz J. Response of the tomato leaf miner *Phthorimaea absoluta* to wild and domesticated tomato genotypes. Pest Manag Sci 2025;81:1345–59. 10.1002/ps.8534 39530398 PMC11821476

[plaf057-B67] Zeist AR, de Resende JT, Perrud AC et al Resistance to *Bemisia tabaci* in tomato species and hybrids and its association with leaf trichomes. Euphytica 2021;217:85. 10.1007/s10681-021-02815-x

[plaf057-B68] Zhang Y, Song H, Wang X et al The roles of different types of trichomes in tomato resistance to cold, drought, whiteflies, and botrytis. Agronomy 2020;10:411. 10.3390/agronomy10030411

[plaf057-B69] Zheng X, Jian Y, Long Q et al SlASR3 mediates crosstalk between auxin and jasmonic acid signaling to regulate trichome formation in tomato. Plant J 2025;121:e70053. 10.1111/tpj.70053 39981944

