# Supplementary material for: Trichome density and herbivore behaviour on tomato is influenced by herbivory, plant age, and leaf surface
Source: AoB Plants. 2025 Oct 7;17(5):plaf057. doi: 10.1093/aobpla/plaf057 (PMC12560162; doi:10.1093/aobpla/plaf057)
Supplement: plaf057_Supplementary_Data [file plaf057_supplementary_data.zip › Supplementary Material 1.docx]

**Supplementary Materials**


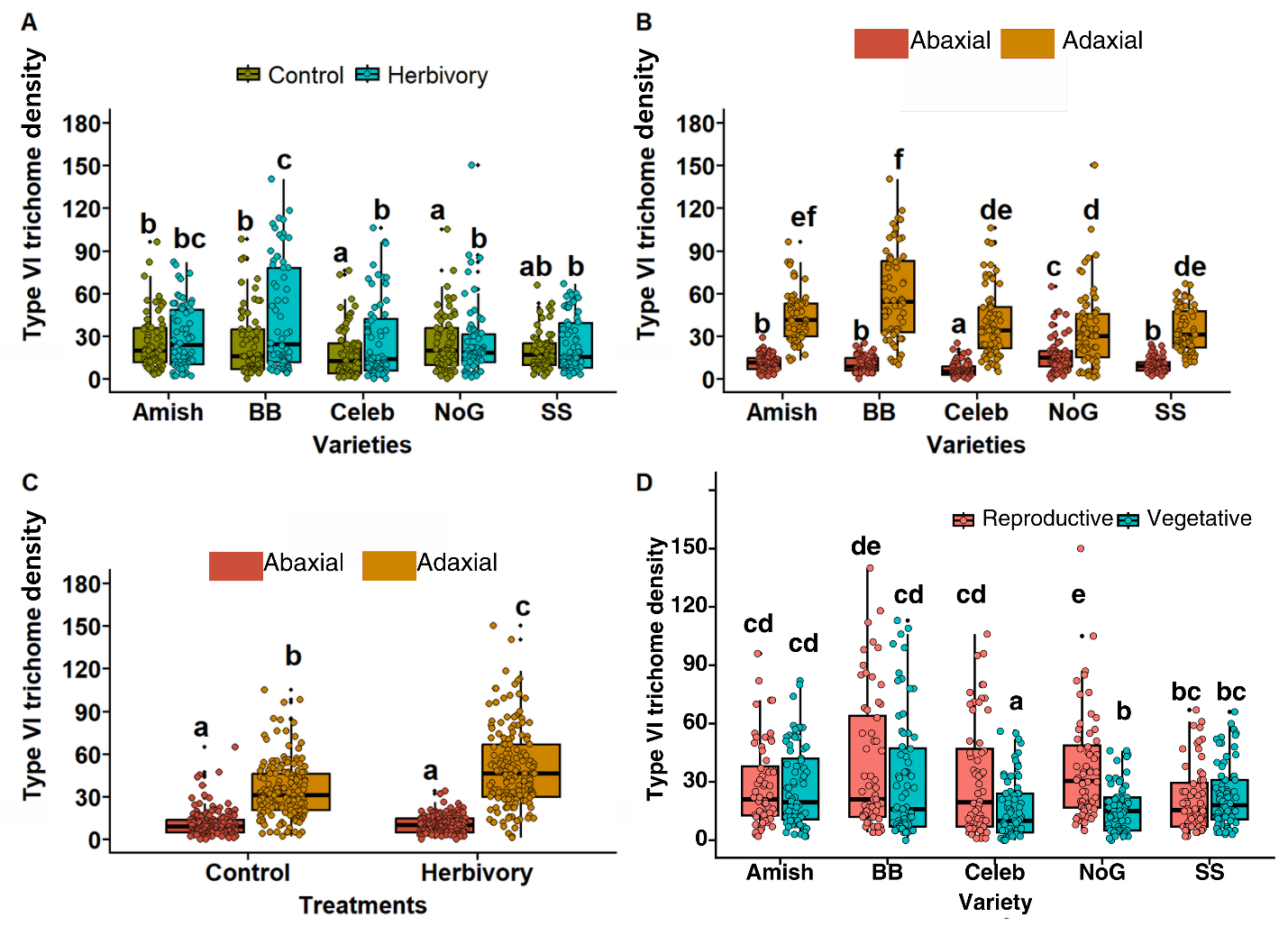
 Fig S1. Two-way interaction on type VI trichome density A) five tomato varieties under control and herbivory treatments B) on the adaxial and abaxial surfaces of tomato leaves across five tomato varieties C) on the adaxial and abaxial surface under control and herbivory treatment D) on leaf surfaces at reproductive and vegetative stages across five tomato varieties. Varieties: Amish- Amish Paste Organic, BB- Big Beef (F1), Celeb -Celebrity (F1), NoG-Nepal Organic, SS- Supersweet 100 F1. The same alphabets indicate no significant differences at the 5 % level of significance.


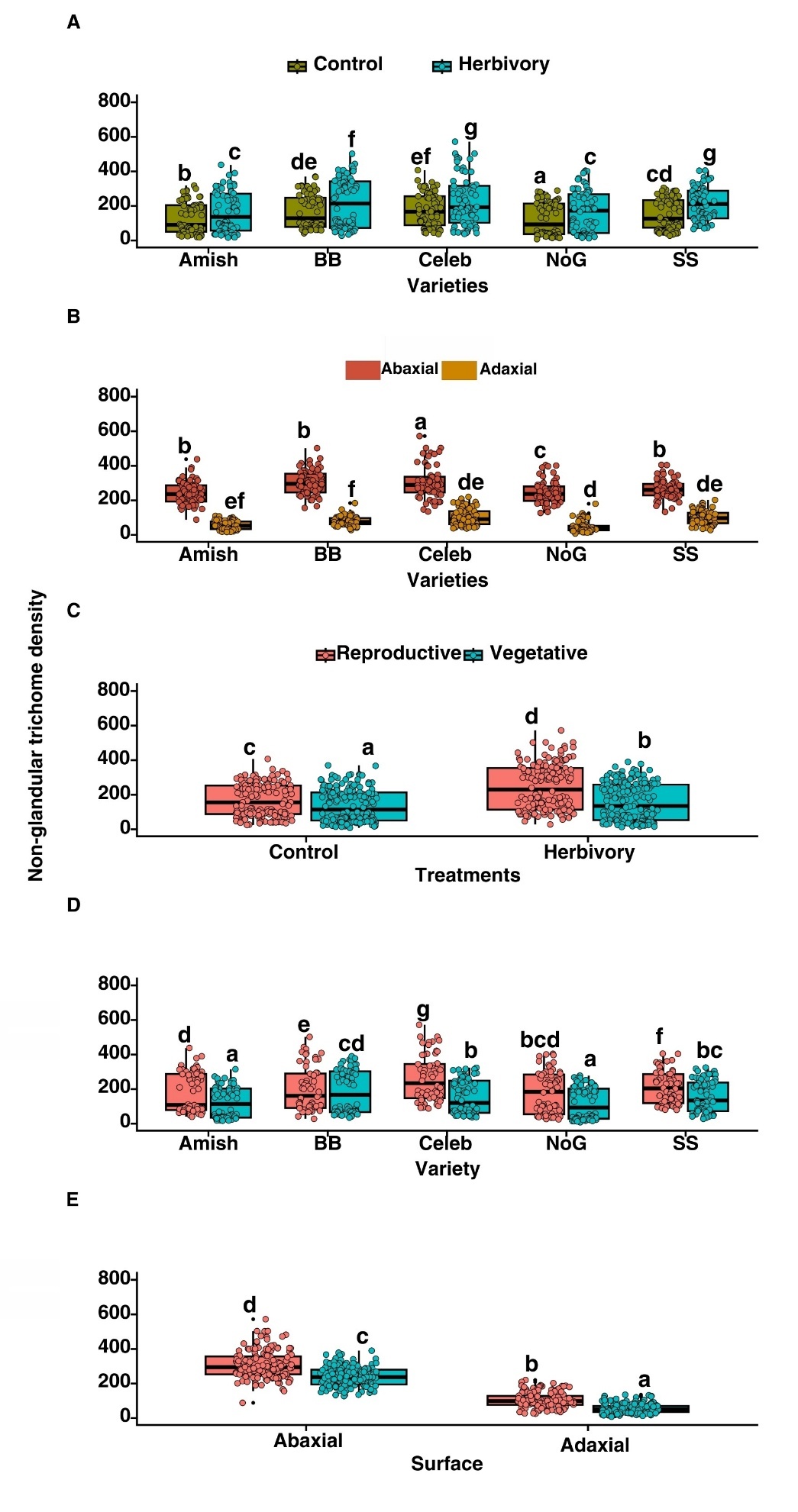


Fig S2. Two-way interaction on non-glandular trichome density. A) Variety × treatment B) Variety × leaf surface C) Treatment × phenological stage D) Variety × plant stage E) Leaf surface × phenological stage. Varieties: Amish- Amish Paste Organic, BB- Big Beef (F1), Celeb -Celebrity (F1), NoG-Nepal Organic, SS- Supersweet 100 F1. The same alphabet indicates no significant differences at the 5 %.


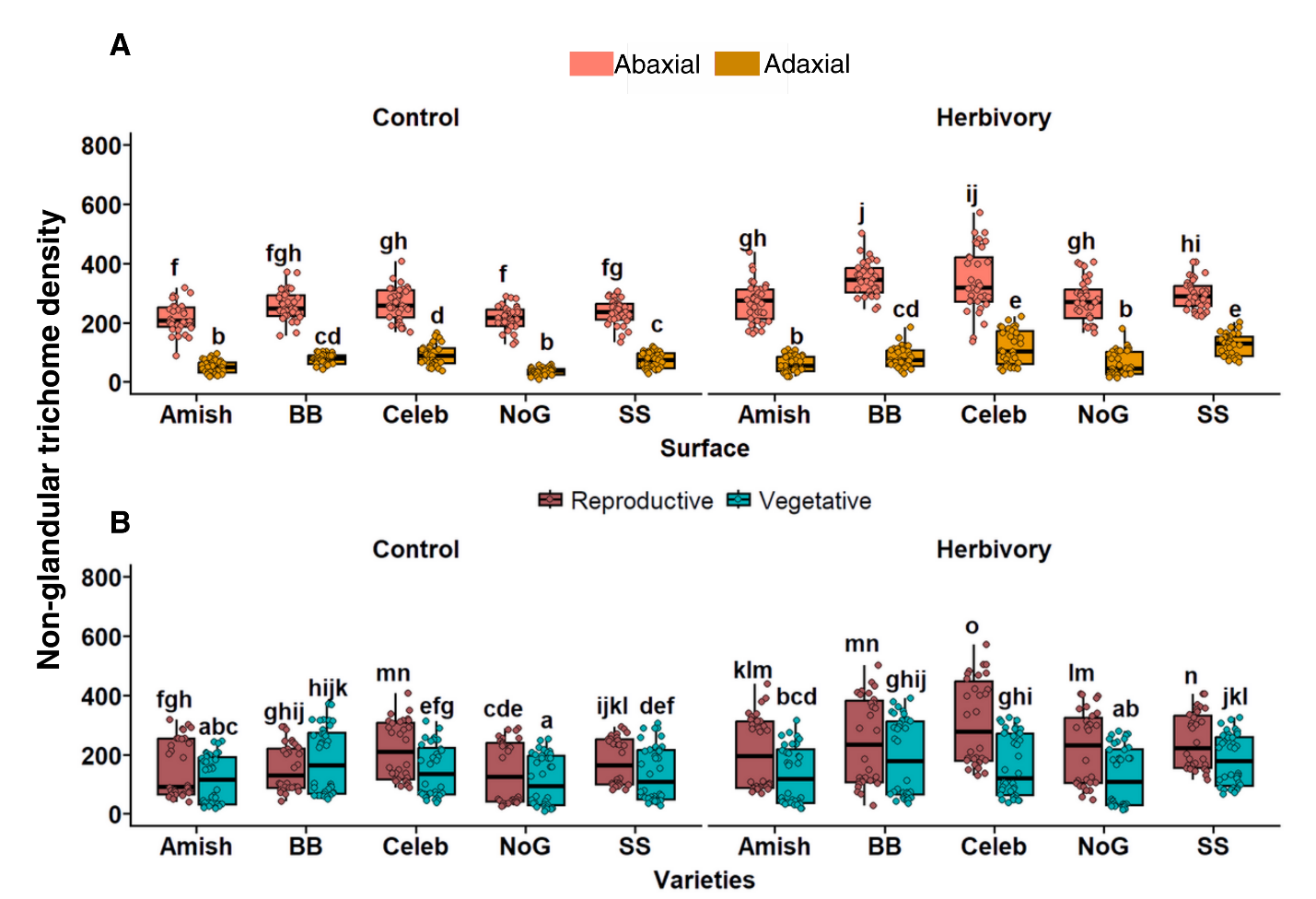


Fig S3. Three-way on non-glandular trichome density A) treatment × variety × surface B) treatment × variety × phenological stage. Varieties: Amish- Amish Paste Organic, BB- Big Beef (F1), Celeb -Celebrity (F1), NoG-Nepal Organic, SS- Supersweet 100 (F1). The same alphabets indicate no significant differences at the 5 % level of significance.


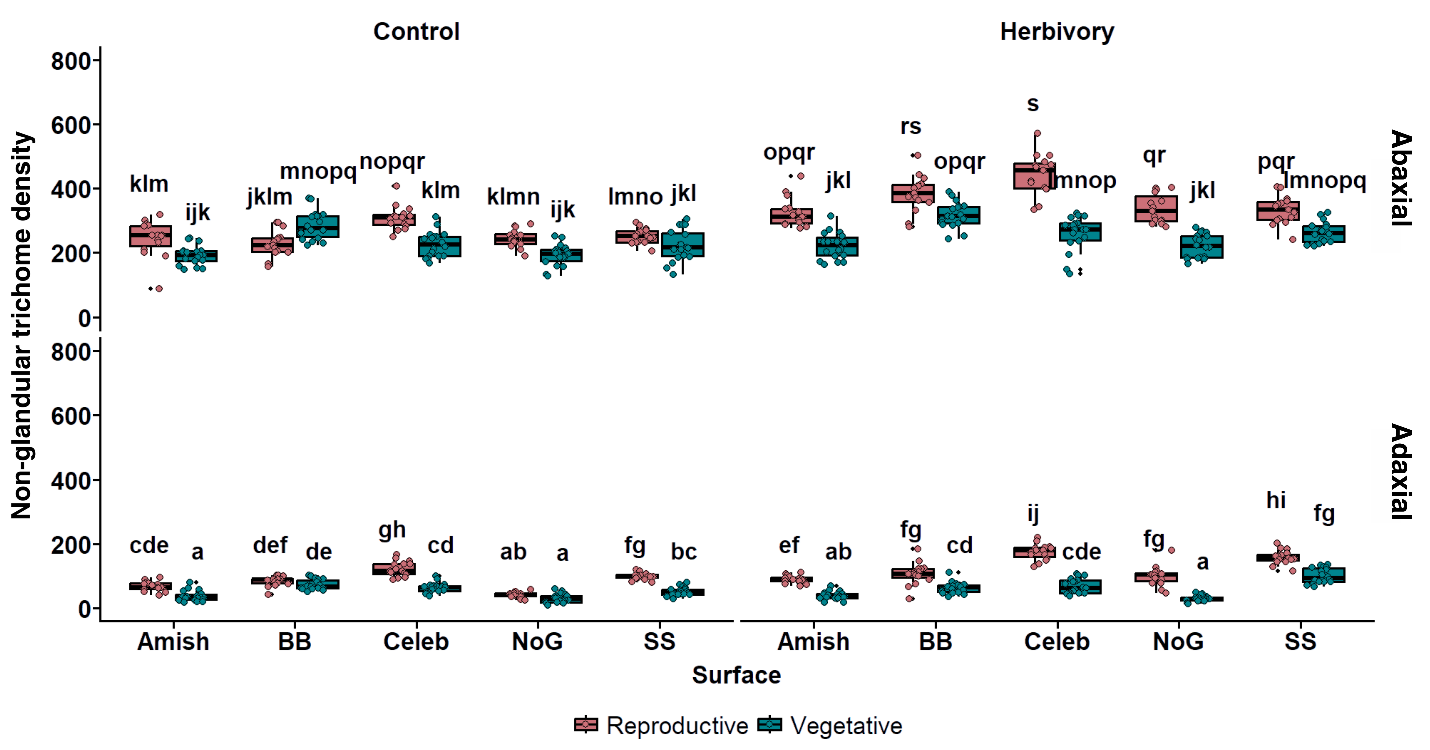
 Fig S4. Four-way interaction on non-glandular trichome density (treatment × variety × surface × phenological stages). Varieties: Amish- Amish Paste Organic, BB- Big Beef (F1), Celeb -Celebrity (F1), NoG-Nepal Organic, SS- Supersweet 100 (F1). The same alphabets indicate no significant differences at the 5 % level of significance.


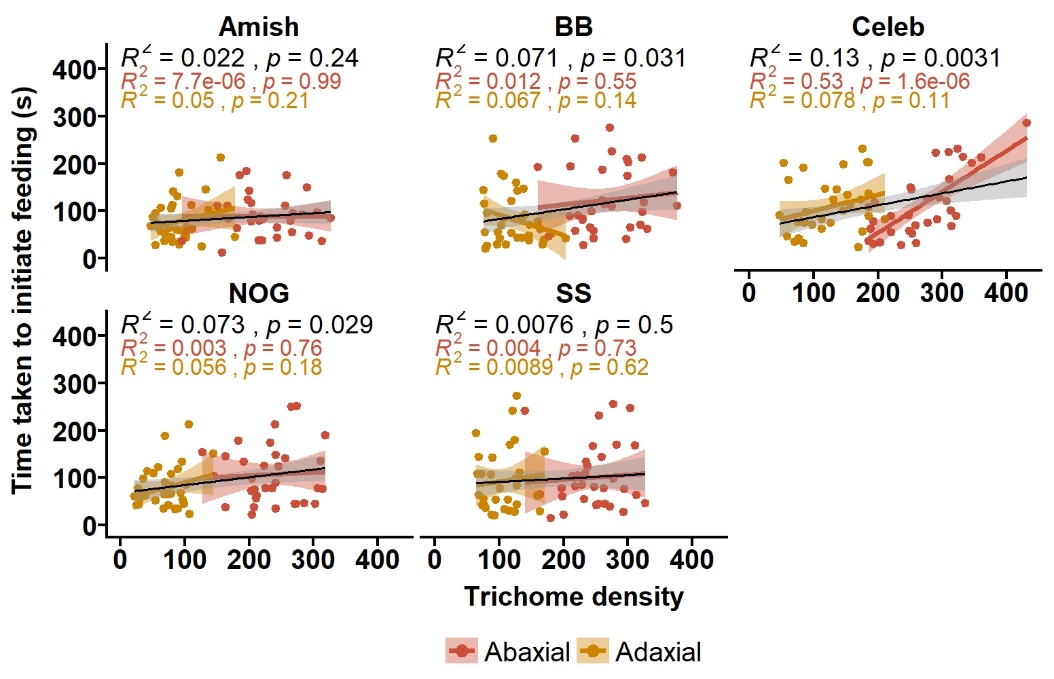


Figure S5. Linear regression analysis for total trichome density by cultivars and leaf surfaces. Varieties: Amish- Amish Paste Organic, BB- Big Beef (F1), Celeb -Celebrity Plus F1, NoG-Nepal Organic, SS- Supersweet 100 (F1).


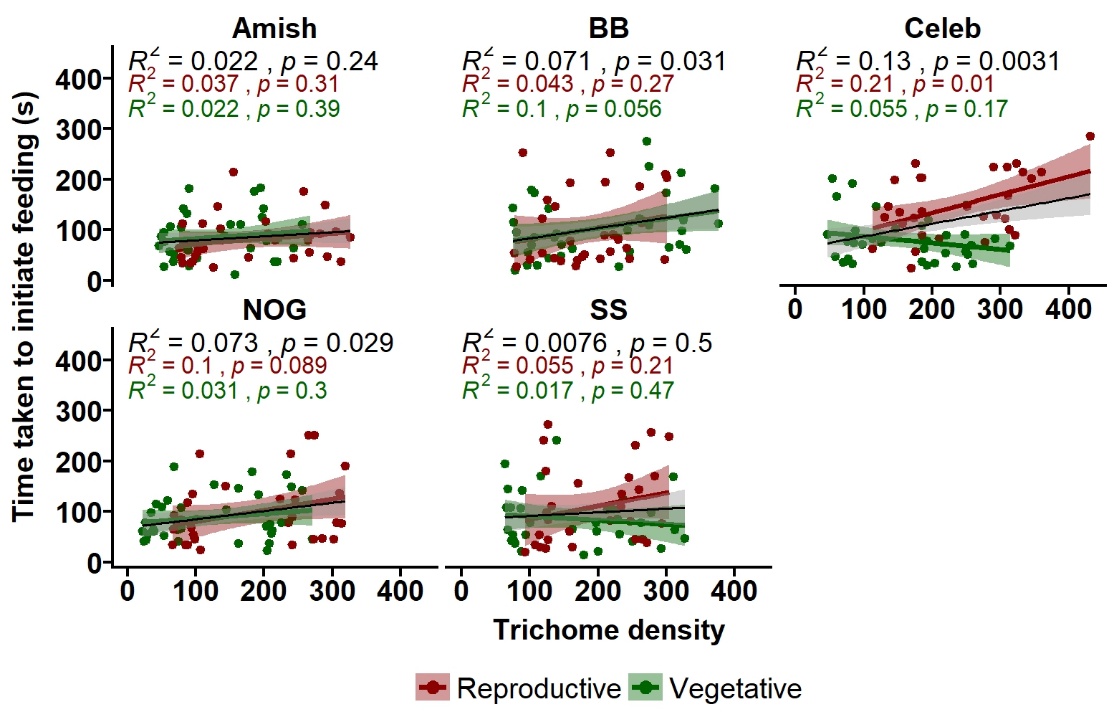


Figure S6. Linear regression analysis for total trichome density by cultivars and phenological stages. Varieties: Amish- Amish Paste Organic, BB- Big Beef (F1), Celeb -Celebrity Plus (F1), NoG-Nepal Organic, SS- Supersweet 100 (F1).


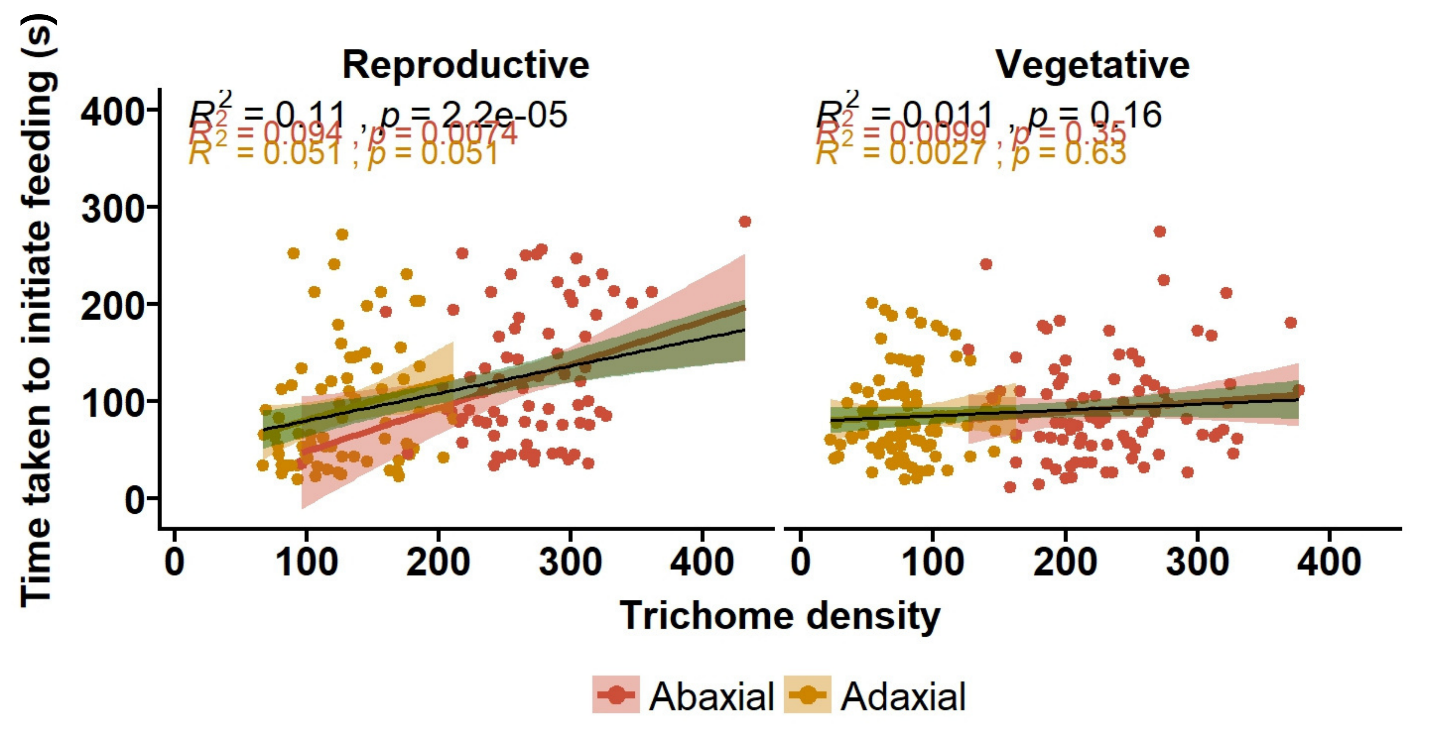


Figure S7. Linear regression analysis for total trichome density by phenological stages and leaf surface.

Table S1. Results of Generalized Linear Model (GLM) with negative binomial regression model for the treatment (herbivory and control), plant phenological stage (Vegetative and reproductive), leaf surface (abaxial and adaxial) and Varieties (Amish Paste organic, Big Beef F1, Celebrity Plus F1, Nepal Organic and Supersweet 100 F1) on type VI glandular trichome and non-glandular trichome density.

| Response/Interaction | Type VI Glandular trichome | | |  | Non-Glandular trichome | | |
| --- | --- | --- | --- | --- | --- | --- | --- |
|  | df | p value | χ^2^ |  | df | p value | χ^2^ |
| Treatment | 1 | <0.001 | 24.0 |  | 1 | <0.001 | 272.00 |
| Variety | 4 | <0.001 | 60.2 |  | 4 | <0.001 | 523.00 |
| Surface | 1 | <0.001 | 1059.3 |  | 1 | <0.001 | 6837.7 |
| Plant stage | 1 | <0.001 | 66.7 |  | 1 | <0.001 | 701.0 |
| Treatment × Variety | 4 | <0.001 | 19.1 |  | 4 | <0.001 | 23.0 |
| Treatment × Surface | 1 | <0.001 | 14.8 |  | 1 | 0.22 | 1.5 |
| Variety × Surface | 4 | <0.001 | 90.2 |  | 4 | <0.001 | 240.0 |
| Treatment × Plant stage | 1 | 0.97 | 0 |  | 1 | <0.001 | 62.8 |
| Variety × Plant stage | 4 | <0.001 | 68.8 |  | 4 | <0.001 | 106.8 |
| Surface × Plant stage | 1 | 0.3 | 1.1 |  | 1 | <0.001 | 151.6 |
| Treatment × Variety × Surface | 4 | 0.91 | 0.9 |  | 4 | <0.001 | 48.0 |
| Treatment × Variety × Plant stage | 4 | 0.14 | 6.8 |  | 4 | <0.001 | 30.3 |
| Treatment × Surface × Plant stage | 1 | 0.72 | 0.1 |  | 1 | 0.17 | 1.9 |
| Variety × Surface × Plant stage | 4 | 0.21 | 5.8 |  | 4 | 0.87 | 1.2 |
| Treatment × Variety × Surface × Plant Stage | 4 | 0.051 | 9.5 |  | 4 | <0.001 | 26.3 |

Table S2. Results of Generalized Linear Model (GLM) with negative binomial regression model for the treatment (herbivory and control), plant phenological stage (Vegetative and reproductive), and Varieties (Amish Paste organic, Big Beef F1, Celebrity Plus F1, Nepal Organic and Supersweet 100 F1) on the densities of total leaf trichome.

| Response/Interaction | DF | P value | χ^2^ |  |
| --- | --- | --- | --- | --- |
| Treatment | 1 | <0.001 | 222.9 |  |
| Variety | 4 | <0.001 | 206.6 |  |
| Plant stage | 1 | <0.001 | 405.9 |  |
| Treatment × Variety | 4 | 0.29 | 4.93 |  |
| Treatment × Plant stage | 1 | <0.001 | 35.9 |  |
| Variety × Plant stage | 4 | <0.001 | 94.5 |  |
| Treatment × Variety × Plant stage | 4 | 0.11 | 7.4 |  |

Table S3. Results of Generalized linear model with Gamma distribution for the tomato varieties (Amish Paste Organic, Big Beef F1, Celebrity Plus F1, Nepal Organic, and Supersweet 100 F1), leaf surface (abaxial and adaxial), and plant phenological stage (Vegetative and reproductive) on the time to initiate feeding by *S. exigua* after placing on leaf surfaces.

| Response | df | p value | χ^2^ |
| --- | --- | --- | --- |
| Variety | 4, 387 | 0.02 | 145.2 |
| Surface | 1, 386 | <0.001 | 141.14 |
| Plant stage | 1, 385 | 0.001 | 137.5 |
| Variety × leaf surface | 4, 381 | 0.29 | 136.1 |
| Variety × Plant stage | 4, 377 | 0.01 | 131.7 |
| Leaf surface × Plant stage | 1, 376 | 0.15 | 131.1 |
| Variety × leaf surface × Plant stage | 4, 372 | 0.35 | 129.5 |
