# Supplementary figures and images for: Trichome density and herbivore behaviour on tomato is influenced by herbivory, plant age, and leaf surface
Source: AoB Plants. 2025 Oct 7;17(5):plaf057. doi: 10.1093/aobpla/plaf057 (PMC12560162; doi:10.1093/aobpla/plaf057)

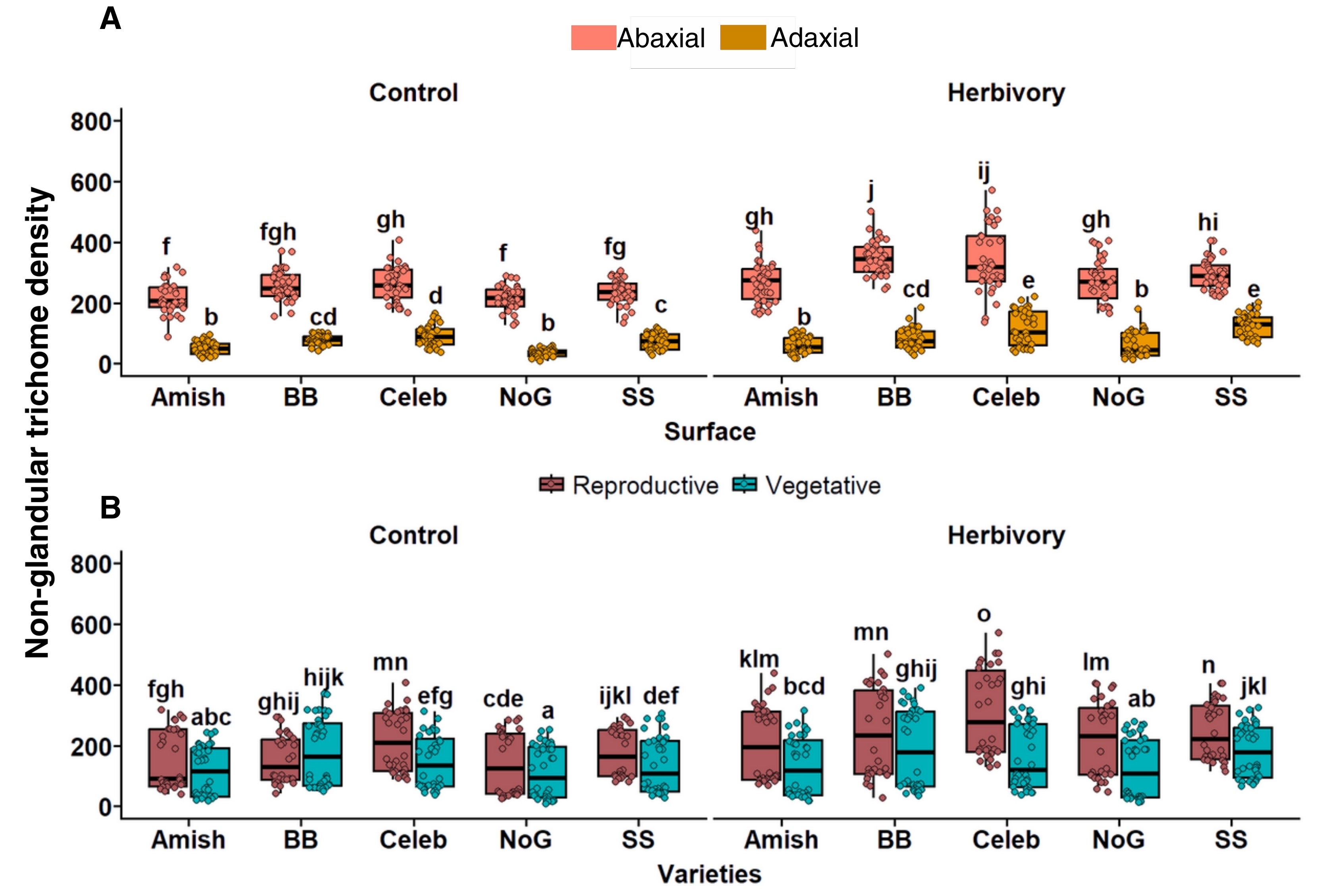

Supplement: plaf057_Supplementary_Data [file plaf057_supplementary_data.zip › Fig S3.jpg]

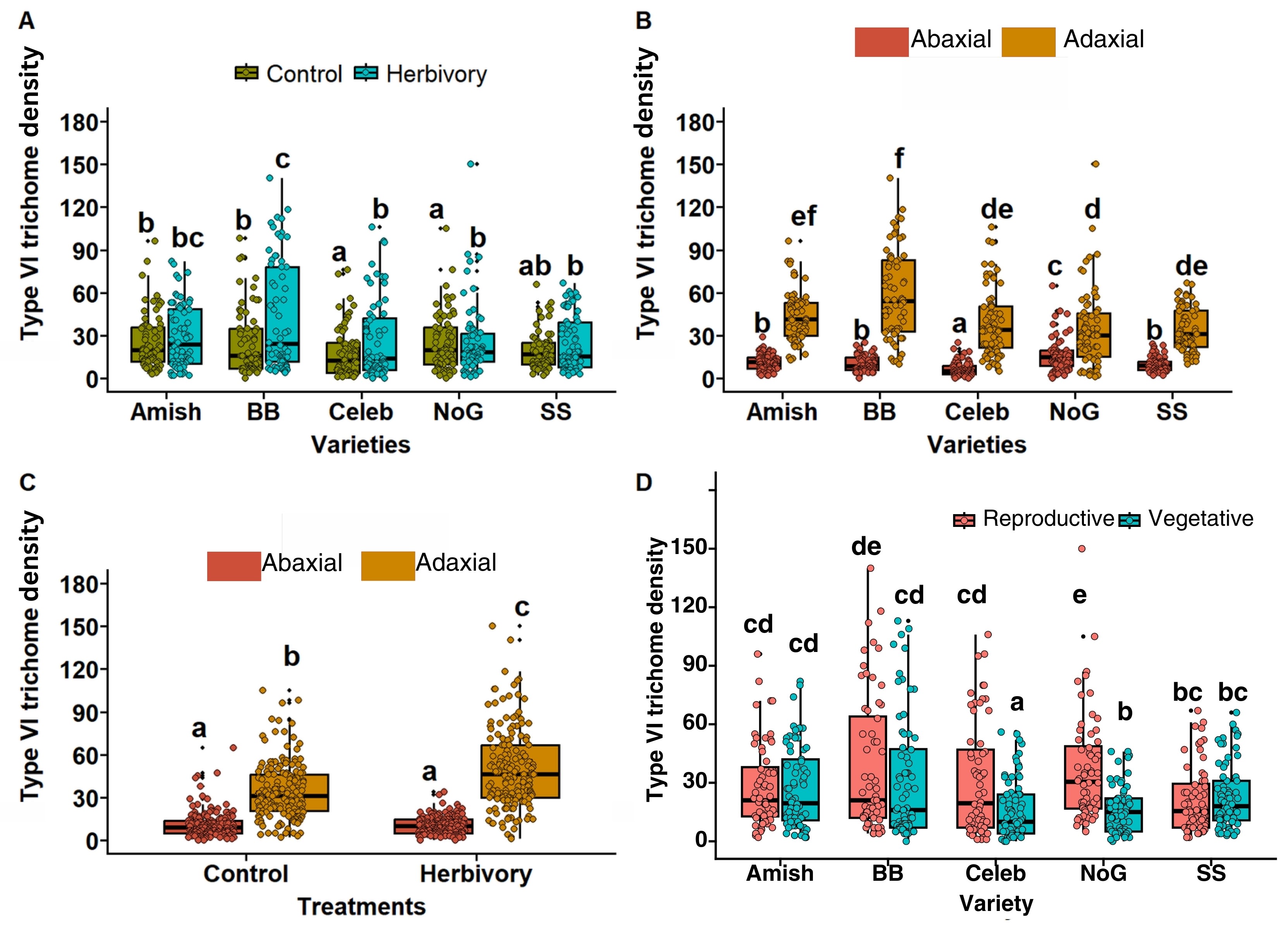

Supplement: plaf057_Supplementary_Data [file plaf057_supplementary_data.zip › Fig. S1.jpg]

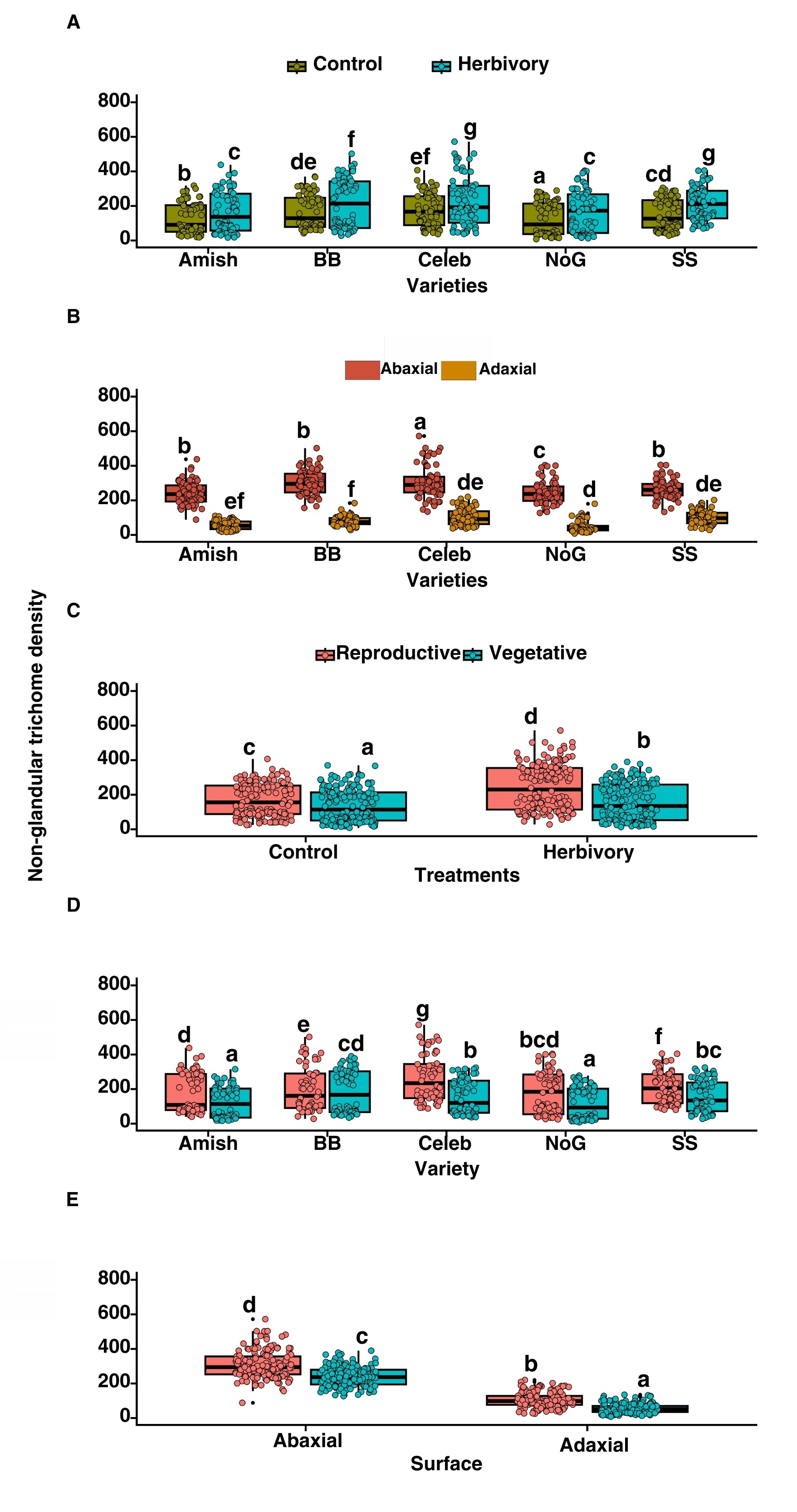

Supplement: plaf057_Supplementary_Data [file plaf057_supplementary_data.zip › Fig. S2.jpg]

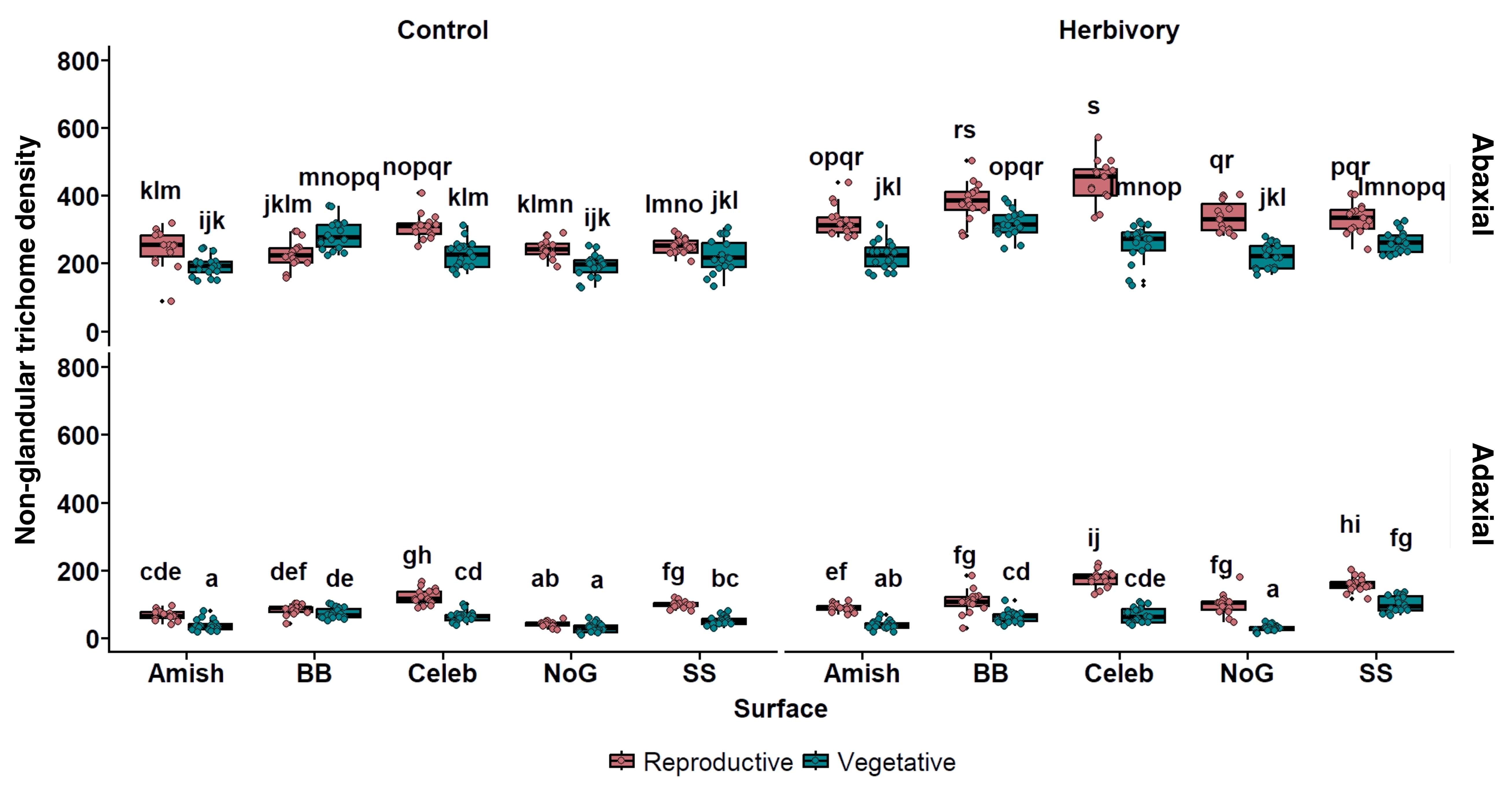

Supplement: plaf057_Supplementary_Data [file plaf057_supplementary_data.zip › Fig. S4.jpg]

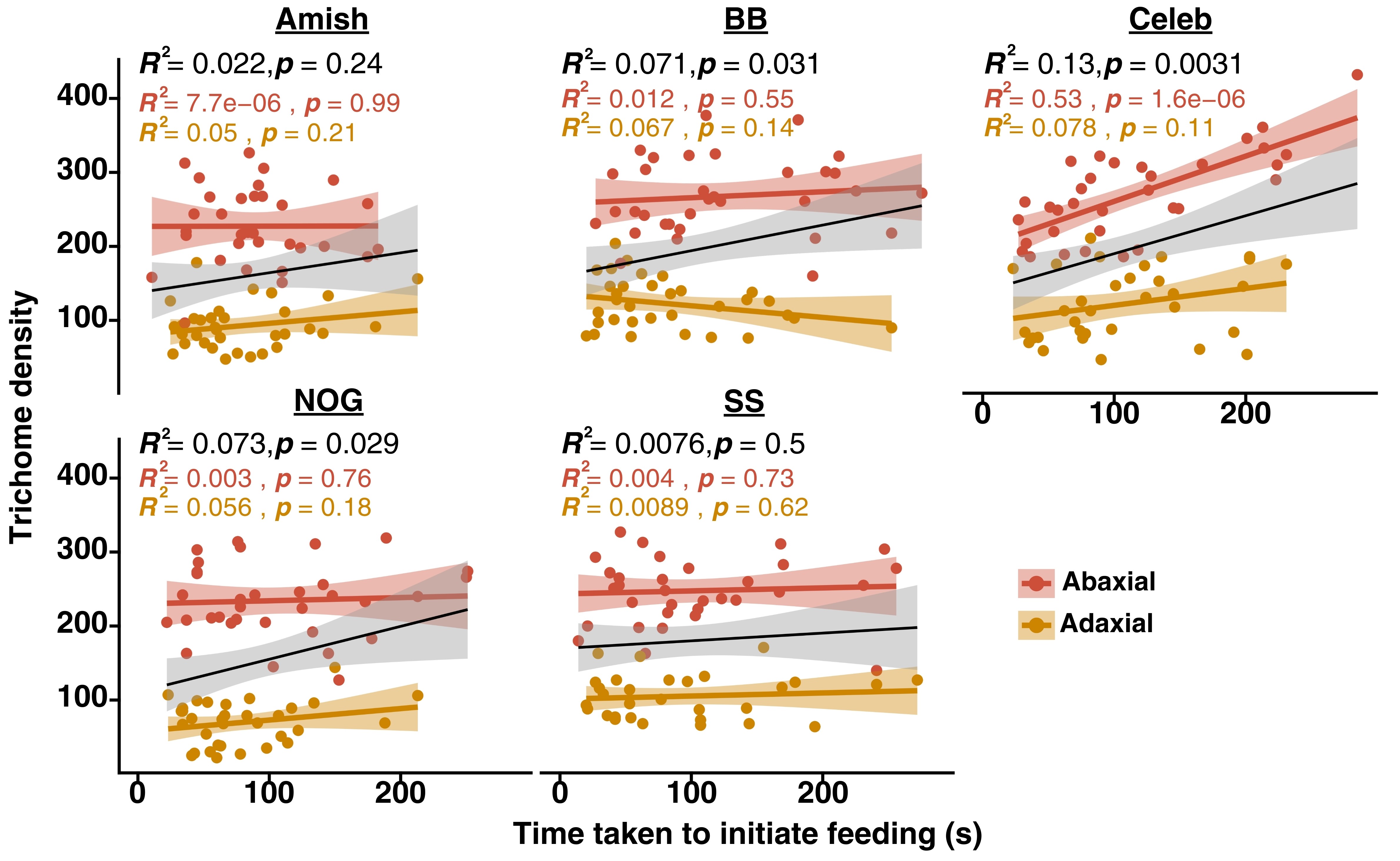

Supplement: plaf057_Supplementary_Data [file plaf057_supplementary_data.zip › Fig. S5.jpg]

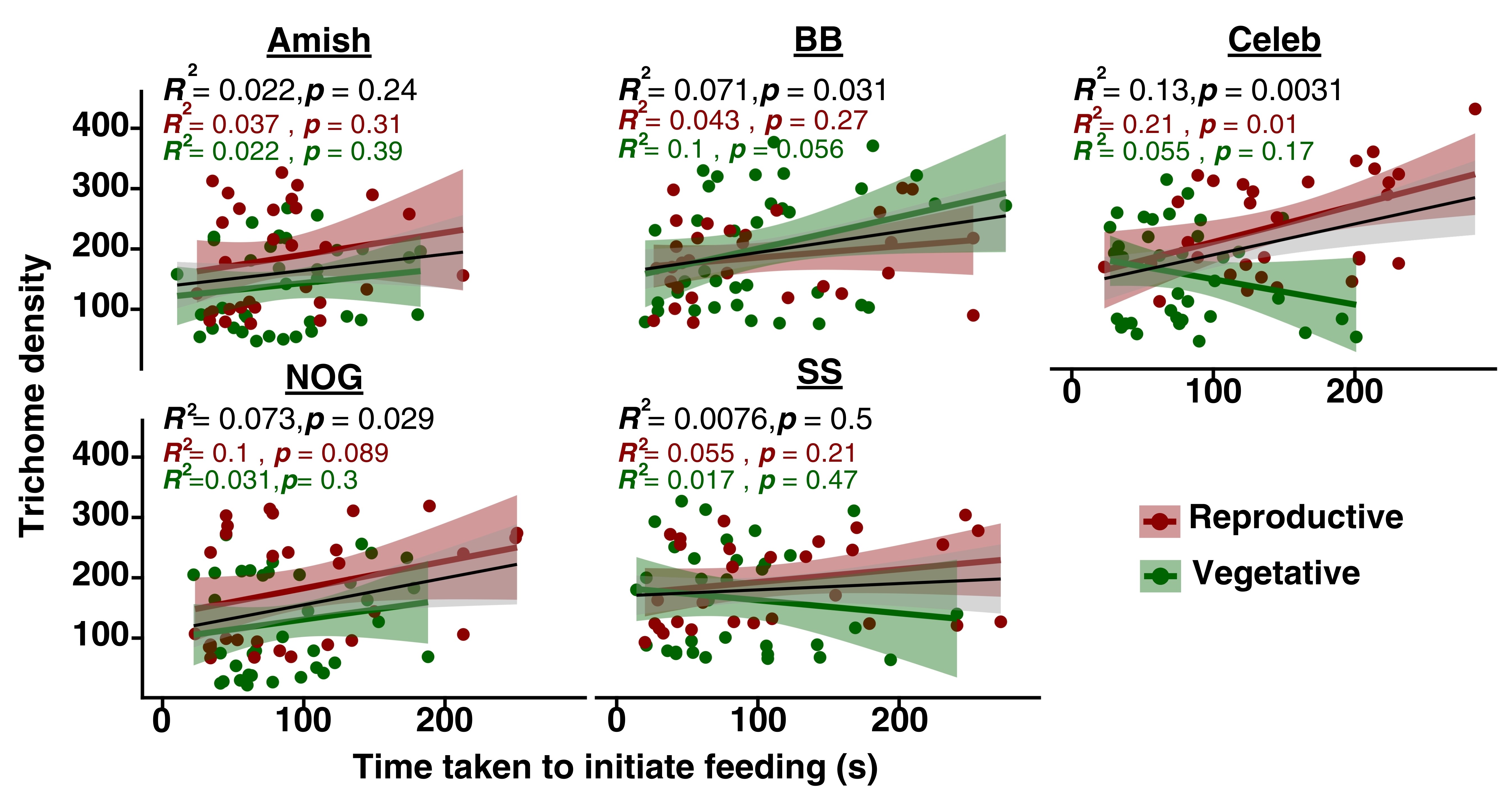

Supplement: plaf057_Supplementary_Data [file plaf057_supplementary_data.zip › Fig. S6.jpg]

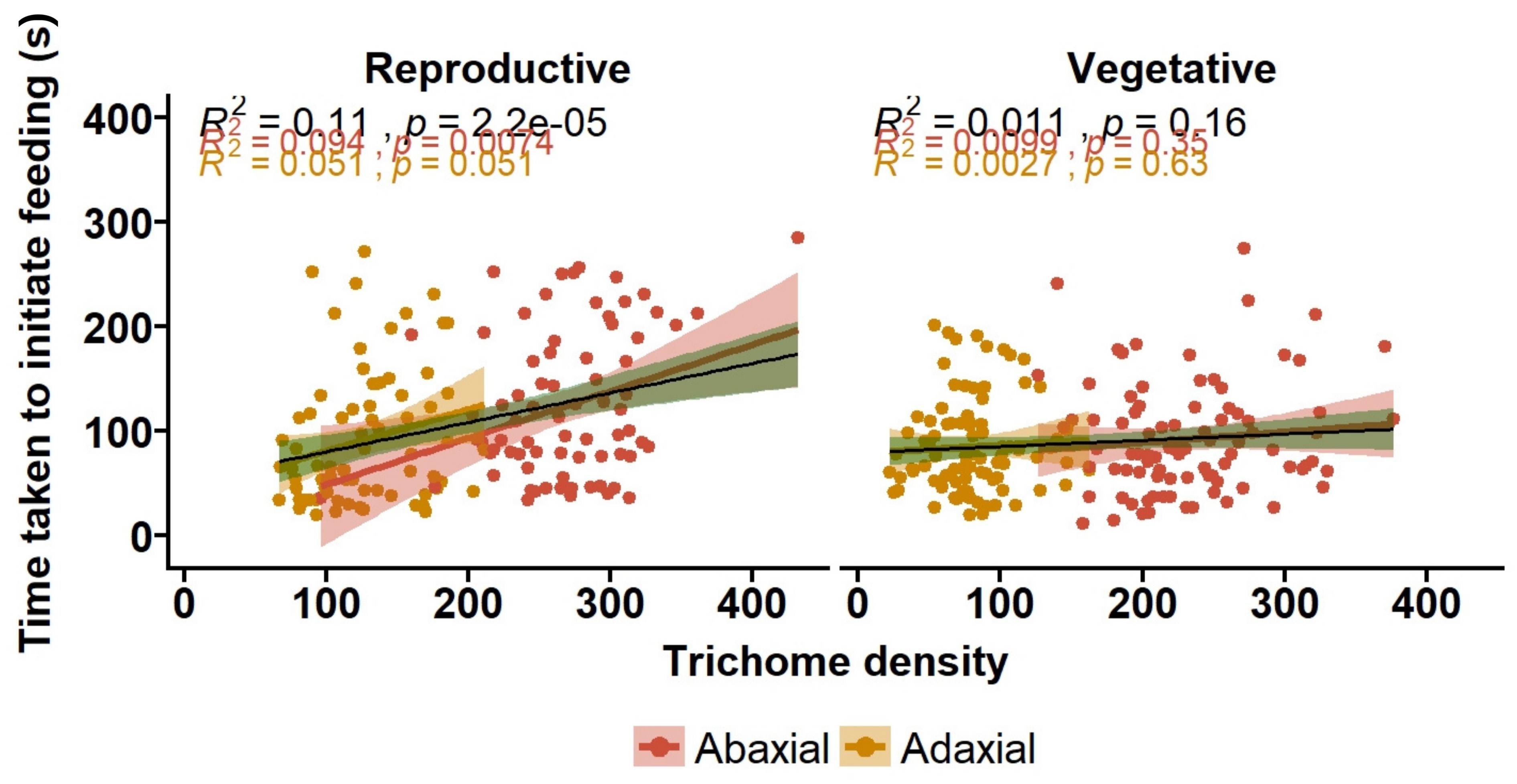

Supplement: plaf057_Supplementary_Data [file plaf057_supplementary_data.zip › Fig. S7.jpg]
